# Absolute protein quantification of the yeast chaperome under conditions of heat shock

**DOI:** 10.1002/pmic.201500503

**Published:** 2016-07-22

**Authors:** Rebecca J. Mackenzie, Craig Lawless, Stephen W. Holman, Karin Lanthaler, Robert J. Beynon, Chris M. Grant, Simon J. Hubbard, Claire E. Eyers

**Affiliations:** ^1^ Centre for Proteome ResearchInstitute of Integrative BiologyUniversity of LiverpoolBiosciences BuildingLiverpoolUK; ^2^ Faculty of Life SciencesUniversity of ManchesterMichael Smith BuildingManchesterUK

**Keywords:** Cell Biology, Chaperone, Heat shock, Label free, QconCAT, *S. cerevisiae*, Selected reaction monitoring

## Abstract

Chaperones are fundamental to regulating the heat shock response, mediating protein recovery from thermal‐induced misfolding and aggregation. Using the QconCAT strategy and selected reaction monitoring (SRM) for absolute protein quantification, we have determined copy per cell values for 49 key chaperones in *Saccharomyces cerevisiae* under conditions of normal growth and heat shock. This work extends a previous chemostat quantification study by including up to five Q‐peptides per protein to improve confidence in protein quantification. In contrast to the global proteome profile of *S. cerevisiae* in response to heat shock, which remains largely unchanged as determined by label‐free quantification, many of the chaperones are upregulated with an average two‐fold increase in protein abundance. Interestingly, eight of the significantly upregulated chaperones are direct gene targets of heat shock transcription factor‐1. By performing absolute quantification of chaperones under heat stress for the first time, we were able to evaluate the individual protein‐level response. Furthermore, this SRM data was used to calibrate label‐free quantification values for the proteome in absolute terms, thus improving relative quantification between the two conditions. This study significantly enhances the largely transcriptomic data available in the field and illustrates a more nuanced response at the protein level.

**Abbreviations**
ChapCAT: chaperone QconCAT
cpc: copy per cell
HS: heat shock
HSF1: heat shock transcription factor‐1
HSR: heat shock response
NG: normal growth
PQC: protein quality control
SRM: selected reaction monitoring
XIC: extracted ion chromatogram

## 1 Introduction

Constantly challenged by their changing environments, most organisms have evolved rapid adaptation responses to external stresses. For example, elevation of temperatures above the optimal growth conditions for *Saccharomyces cerevisiae* activates a protective transcriptional program known as the heat shock response (HSR). The attendant changes in physiology and metabolic flux support the maintenance of growth up to temperatures around 42°C 1. Nevertheless, such a temperature shift can have profound effects on the proteome, since protein stability is sensitive, fluctuating between aggregation‐prone, near‐native conformational states and native folded states. An increase in thermal energy can shift the conformational equilibrium towards more aggregation‐prone states in which the exposed hydrophobic regions of the unfolded proteins interacts with one another leading to protein aggregation 2.

**Significance of the study**

We demonstrate that the majority of *S. cerevisiae* chaperones are upregulated in response to heat shock, with an average two‐fold change. Those proteins that become significantly upregulated are direct gene targets of heat shock transcription factor‐1, with known roles in the protection of misfolded proteins from further aggregation, protein refolding, including those from perivacuolar insoluble deposit and juxtanuclear compartments, and ultimately degradation via the proteasome. Furthermore, we demonstrate that MaxLFQ SRM‐normalisation approaches better predict the fold changes occurring between normal growth (NG) and heat shock (HS) treated cells than simply comparing MaxLFQ values. This dataset may be used to investigate the chaperone‐client ‘interactome’ in response to heat shock, permitting insight into the chaperone pathways mediating cellular protection against misfolded protein.

Molecular chaperones are important in mitigating against such aggregation. They play a vital role in the folding and trafficking of protein molecules in the cellular stress response, as well as contributing to cellular homeostasis under normal conditions 1, 2, 3, 4. Chaperone activity is dependent on appropriate interactions with their client proteins, in addition to protein co‐factors and ribosomes, assisting folding of newly synthesised polypeptide chains and minimising protein misfolding and aggregation. Together, these chaperone‐protein interactions form the ‘chaperome’ network. The synthesis of many chaperones increases as a result of the HSR to counteract protein misfolding and aggregation and prevent cellular disorder 4, 5; this is the origin of the term ‘Heat Shock Proteins’ (HSPs).

Elevated temperature increases the fluidity of *S. cerevisiae* cell membranes, which translates into the specific activation of heat‐sensing Ca^2+^ channels and a downstream signalling cascade resulting in the activation of heat shock transcription factor‐1 (Hsf1), the primary modulator of the HSR 6, 7. Hsf1 binds to the heat shock element contained within the promoters of its target genes which commonly function as chaperones 8. In addition to activation of these specific protein factors, the HSR includes: accumulation of the storage carbohydrates trehalose and glycogen – a response activated by the transcription factors Msn2/4; transient arrest of the cell cycle at the G1 stage due to inhibition of the cyclins Cln1 and Cln2 by Hsf1, and thermotolerance against future stress, achieved via activation of the Pkc1‐MAP kinase pathway (the cell wall integrity pathway) 9, 10. During HSR, the primary role of chaperones is considered to be the protection of the hydrophobic surfaces of misfolded and aggregated proteins. Terminally misfolded proteins may be directed to the ubiquitin‐proteasome pathway for degradation, whilst others may be unfolded and later refolded when favourable conditions return. Therefore, without chaperone upregulation, cellular protection during and recovery after heat shock is not possible. As such, chaperones are fundamental cellular effectors of the HSR.

Previous proteomic and transcriptomic studies have characterised chaperone upregulation in response to various stress conditions. However, proteomics analyses have generally used relative quantification rather than defining changes in absolute protein levels. Published studies have also typically been limited to a subset of the chaperones/proteome, or focussed on transcriptional responses at the mRNA level. Proteomic studies have typically used SILAC approaches, pulse labelling with ^35^S‐methionine and semi‐quantitative western blots to measure the proteome directly, whilst northern blots and DNA microarrays have inferred transcriptome changes 11, 12, 13, 14, 15, 16, 17, 18. Although we have previously quantified absolute protein abundance (copies per cell) for over 50 chaperones, the study was only performed under normal, chemostat growth conditions. Regardless, using a simple model and known substrate interactions 19 we estimated that ∼62% of total protein folding flux in the chemostat‐grown cell is chaperone‐mediated 20.

Given that prior proteomic studies of the *S. cerevisiae* HSR have either been incomplete, or ‘relative’ in nature, we have extended our previous QconCAT SRM‐based absolute quantification study 20 to chaperones under both normal batch growth (NG) and heat shock (42°C, 30 min) (HS) conditions.

We have also examined the potential gains of increasing the number of internal reference quantification peptides (‘Q‐peptides’ 21, 22) selected per protein from two to five. In parallel, we have also performed label‐free quantification of the attendant proteome under NG and HS, to assess changes to substrate protein levels. These studies agreed well with the respective absolute abundances, and we were able to calibrate the label‐free data using a MaxLFQ SRM‐normalisation approach, similar to that published previoulsy 23. Collectively, the data define the protein‐level HSR in absolute terms for the first time, offering new insights into cellular proteostasis at the molecular level.

## 2 Materials and methods

### 2.1 Design of chaperone QconCATS (ChapCATs)

The sequences of the 63 known chaperones in *S. cerevisiae* were subject to in silico tryptic digestion, and the limit peptides analysed for suitability as Q‐peptides for QconCATs (which we term here as ‘ChapCAT’) according to criteria previously outlined 21, 24, 25. Briefly, peptides must be sequence‐unique to the protein and proteome under investigation, and not known to be post translationally modified according to dbPTM (http://dbptm.mbc.nctu.edu.tw/) 26. Propensity for the peptide to undergo missed cleavage in the native protein sequence was evaluated using MC:pred (http://king.smith.man.ac.uk/mcpred/), recording scores for both the *N*‐terminal and *C*‐terminal bond 27. Likelihood of detection in a liquid chromatography‐electrospray ionisation mass spectrometry (LC‐ESI‐MS) experiment was assessed using CONSeQuence 28, available at http://king.smith.man.ac.uk/CONSeQuence/. Candidate peptides were omitted if their sequence contained any of the following features: dibasic sequences; Asn‐Gly motifs or contiguous Gln (2–5) residues; < five amino acids; or were reported to have a PTM. From the Q‐peptides used previously 20, 31 were retained in the final Q‐peptide set. A combination of CONSeQuence score and MC:pred scores allowed us to rank potential Q‐peptides, with the top five Q‐peptides (where possible) selected per chaperone protein. For Hsp31, Sno4 and Hsp33, no unique quantotypic peptides (i.e. fully tryptic peptides suitable for use as quantification standards) were identified; non‐unique Q‐peptides were therefore selected representing the summed protein group. Non‐unique but potential Q‐peptides were also observed for the protein pairs Ssa1:Ssa2 and Ssb1:Ssb2 and selected as quantification standards owing to few unique alternatives; both unique and non‐unique Q‐peptides were used to improve quantification reliability.

Q‐peptides were assigned to a ChapCAT such that an individual ChapCAT targeted chaperones in the same general chaperone class (defined as per 20). A total of 10 ChapCATs were designed, each targeting six to eight chaperones and containing 25–37 Q‐peptides. The constituent Q‐peptides were concatenated in silico within a ChapCAT for maximal likelihood of completion of tryptic cleavage, determined using MC:pred 27. Concatenated Q‐peptides were used to direct the design of a gene, codon‐optimised for expression in *Escherichia coli* (PolyQuant GmbH, Germany).

### 2.2 Expression and purification of ChapCATs in *E. coli*

ChapCAT proteins were expressed in *E. coli* and purified as previously described 24 with only minor alterations (see Supporting Information). Expression of some ChapCATs required additional optimisation of expression conditions (see Supporting Information Table S1 and Figs. S1 and S2), whilst some, as noted, required peptide rearrangement and re‐synthesis.

### 2.3 Preparation of *S. cerevisiae* samples and ChapCAT digestion

*S. cerevisiae* (EUROSCARF accession number Y11335 BY4742; *Mat ALPHA*; *his3Δ1*; *leu2Δ0*; *lys2Δ0*; *ura3Δ0*; *arg3::KanMX4*) was grown in C‐limited F1 medium 29, such that 10 g/L of glucose was the only carbon source. To meet auxotrophic requirement of the strain, 0.5 mM arginine and 1 mM lysine were introduced into the F1 medium. A 5 mL pre‐culture inoculated with a single *S. cerevisiae* colony was incubated at 30°C for 24 h prior to inoculation of eight biological replicates of 50 mL F1 medium. Samples were grown overnight (30°C) to an OD_600_ of 2. To prepare the HS samples, four of the eight replicates were removed and placed in a water bath with shaking at 42°C for 30 min. Subsequently, individual samples were aliquoted (15 mL) and cell counts recorded using an Auto M10 Cellometer® (Nexcelom, Manchester) prior to centrifugation (4000 rpm, 4°C, 10 min). For label‐free quantification, protein concentration was determined by Bradford assay and equivalent amounts analysed. Extraction of proteins, addition of ChapCAT and subsequent tryptic digestion was carried out as previously described 20, 30. To check complete digestion of yeast and to quantify ChapCAT, each digest was analysed by LC‐MS using a nanoAcquity UPLC^TM^ system (Waters, Manchester) coupled to a Synapt^TM^ G2‐Si mass spectrometer (Waters, Manchester) in MS^E^ mode. The data were searched against a sequence database created from the sequences of ChapCAT001 to ChapCAT008, with fixed modifications for carbamidomethylation of cysteine and ^13^C_6_ labelling of arginine and lysine using ProteinLynx Global Server v2.5 (Waters). The ChapCAT was quantified via integration of the extracted ion chromatogram (XIC) of the ChapCAT heavy glu‐fibrinopeptide standard (*m/z* 788.8) compared to exogenously added light internal standard glu‐fibrinopeptide (*m/z* 785.8) 20. To determine digestion efficiency in both the ChapCAT standard and analyte, yeast aliquots containing the equivalent of 25 000 000 cells and 22.5 pmoles of ChapCAT were subjected to tryptic digestion as previously described 20, 31. At 0, 1, 2, 5, 10, 20, 50, 120, 240, 270 (enzyme top up) and 1230 min, a 10 μL portion of sample was removed and incubated with 10 μL of 5% (v/v) TFA to terminate proteolysis.

### 2.4 Mass spectrometry and data analysis

The seven most intense product ions generated following collision‐induced dissociation (CID) were selected as transitions prior to unscheduled analysis by selected reaction monitoring (SRM). Each digested ChapCAT in a NG background was analysed using a nanoAcquity UPLC^TM^ system (Waters, Manchester) coupled to a Xevo^TM^ TQ(‐S) triple quadrupole mass spectrometer (Waters, Manchester). Based on these analyses, the three transitions with the greatest S/N ratio (as calculated in Skyline 32) were selected for the final scheduled SRM analysis and quantification (Supporting Information S2) 20. Scheduling was done in three minute windows around the retention time of the peptide, using the same three transitions for both NG and HS extracts. For the digestion time course, a scheduled SRM experiment was performed on sample volumes equivalent to 200 000 cells using a nanoAcquity UPLC^TM^ system (Waters, Manchester) coupled to an Xevo^TM^ TQS triple quadrupole mass spectrometer (Waters, Manchester). Transitions and scheduling windows were identical to those used for absolute quantification. For final protein quantification, sample volumes containing the equivalent of 200 000 cells with 0, 0.2, 2 or 20 fmol of ChapCAT were loaded. The sample closest to a 1:10 ratio between Q‐peptide and analyte XIC was selected for final quantification. Fresh digests (1 μg) of the same NG and HS yeast samples (not containing ChapCATs) were subject to label‐free quantification by data‐dependent acquisition (DDA) using a Dionex UltiMate^TM^ 3000 HPLC system coupled to a Q‐Exactive HF mass spectrometer with an EASY‐Spray^TM^ column and source (ThermoScientific, Hemel Hempstead) in an unfractionated experiment (full details in Supporting Information). Following a full MS scan between *m/z* 350 and 2000 (mass resolution of 60 000 FWHM at *m/z* 200), a data‐dependent top‐16 method MS^2^ analysis was performed with a target value of 1×10^5^ ions determined with automatic gain control. Precursor ions were isolated with an isolation window of *m/z* 1.2, with scans acquired at a mass resolution of 30 000 FWHM at *m/z* 200 and dynamic exclusion of 20 s. Three biological replicates were analysed for NG samples (NG1, NG3 and NG4) and four biological replicates (HS1, HS2, HS3 and HS4) for the HS samples.

### 2.5 Data processing and analysis

To determine digestion efficiency of the Q‐peptides in the standard and the related endogenous analyte peptide, data acquired for each time point was processed with Skyline and a report detailing the total peak area for each peptide at each time point exported. The pseudo‐first order rate kinetics were modelled using the ‘nls’ function in the statistical software package R, as was previously carried out 31. For each Q‐peptide, the rate constant (*k*) was determined for both the standard and analyte, allowing calculation of the respective digestion half‐lives (*ln2/k*). Digestion was deemed complete at five half‐lives (full details in Supporting Information).

mProphet 33 was used to determine peak areas for both the unlabelled target peptides and isotope‐labelled ChapCAT Q‐peptide internal standards; copies per cell values were calculated using the measured area ratios and the known quantities of Q‐peptides. Production of decoy transitions and the subsequent quantification workflow is described in previous literature 20. To avoid known issues with peak group detection, an in‐house script was developed that set a retention time window +/– 30 s in silico either side of the maximum peak intensity for the peak group, through curation of the merged target and decoy *.mzXML* files (see Supplementary Information). The subsequent *.mzXML* files were then processed as previously described 20.

For label‐free quantification, acquired data were processed with MaxQuant (v1.5.2.8) 34 with peptides identified using the Andromeda search engine 35, searching the entire *S. cerevisiae* protein sequence database (canonical and isoform *.fasta* downloaded from UniProt ‐ http://www.uniprot.org/downloads/, accessed April 2015 containing 6721 entries), additional to a reverse decoy database and a database of known contaminants as available within the MaxQuant software. MaxQuant default search parameters were used, specifying two missed cleavages and LFQ minimum ratio count set to one. Additionally, the ‘requantify’ and ‘match between run’ options were selected. The ‘proteinGroups.txt’ file was then manually filtered such that proteins had to be observed with a non‐zero MaxLFQ intensity 36 in at least three biological replicates and quantified using at least two unique peptides. Protein MaxLFQ intensities determined via peptides matched to protein groups were not accepted. Final MaxLFQ intensities for a protein were calculated as the median MaxLFQ intensity across biological replicates for samples obtained under the same growth condition. Protein identifications that passed a 1% FDR against the decoy database were deemed true positive matches and the corresponding Q‐values for each protein identified recorded.

## 3 Results and discussion

### 3.1 Design, expression and purification of ChapCATs

Using the QconCAT methodology, 10 ChapCATs were designed and constructed targeting 63 known chaperones in *S. cerevisiae*. Each ChapCAT contained Q‐peptides designed to quantify chaperones belonging to the same chaperone class according to Gong et al. 19. Where possible, up to five Q‐peptides were selected to target each chaperone. However, for 15 of the 63 chaperones fewer candidate quantotypic peptides passed all the quality control steps; thus nine chaperones were targeted by four Q‐peptides, three chaperones were targeted by three Q‐peptides, two chaperones (Ssb1 and Ssb2) were targeted by only two Q‐peptides, with Ssa1 targeted by a single (unique) Q‐peptide. For the Hsp70s in particular, options are limited by the high sequence similarity between paralogues, restricting peptide choice, although we were able to quantify most proteins using unique Q‐peptides. With the exception of a single ChapCAT (ChapCAT010), for which expression was unsuccessful even after reshuffling of the Q‐peptides within the expression construct, all ChapCATs yielded isotope‐labelled protein following expression in *E. coli* (example in Supporting Information Figs. 1 and 2). Although ChapCAT009 expressed, levels were very low and we elected not to continue with this ChapCAT standard. As such, ChapCAT001 to ChapCAT008 were successfully expressed and purified to a quantifiable amount for MS analysis, targeting 49 chaperones.

### 3.2 Absolute quantification of targeted chaperones

In order for a peptide to be quantified and copy per cell (cpc) values defined, it must be observable in both the heavy‐labelled standard (ChapCAT) and light (unlabelled) native yeast sample. To determine Q‐peptide suitability for protein quantification we refined our previous classification system 20, 22; as before, peptides observed in both labelled (heavy) ChapCAT standard and (light) yeast analyte were classified as ‘A’ peptides; peptides observed solely in the ChapCAT standard – the native yeast peptide being presumably below the limit of detection was classified a ‘B’ peptide; whilst peptides not observed in either the ChapCAT or yeast samples were classified as class ‘C’. We further separated ‘B’ Q‐peptides into two subclasses: ‘B1’ peptides had a low signal in the light channel and therefore lay below the limit of detection whilst for ‘B2’ peptides, the light signal failed to pass mProphet's 1% FDR threshold in comparison with the decoy transitions. We used ‘B1’ and ‘B2’ peptides only to estimate the limit of detection. For each peptide, we selected the lowest ChapCAT concentration with at least a 10:1 S/N ratio and used the respective concentration to estimate the maximum number of copies per cell that could be quantified. The average limit of detection is 700 and 2300 cpc for ‘B1’ and ‘B2’ Q‐peptides, respectively (Supporting Information Table S3). This reflects the features discussed earlier; ‘B1’ peptides are deemed too low to quantify in terms of defining cpc value, whilst ‘B2’ peptides are potential false positives and whose peptide ion signal may contain contaminants.

We also further refined the classification scheme for ‘A’ Q‐peptides, by considering digestion properties of both analyte and standard within the time frame allocated (20.5 h). Accurate quantification presumes complete digestion, or very similar digestion kinetics between the standard and the analyte. A digestion time course was used to estimate the first‐order rate constant, and complete digestion >97% was considered to have occurred at five half‐lives. Peptides that were not deemed complete were classified ‘A2’; eight such peptides were identified under NG or HS conditions and thus were not considered for quantification. As a final quality control step, the transition profiles for all ‘A’ peptide quantifications with robust coefficient of variance (rCV) in excess of 30 were examined manually using Skyline 32. The median peptide rCV was 10.55 and 14.64 for NG and HS conditions, respectively. If the signal intensity order of SRM transitions was inconsistent between heavy and light peptide pairs, or mProphet was judged to have incorrectly selected the peak group, these peptides were also classed as ‘A2’ and not considered at the protein level (additional parameters discussed in Supporting Information Figs. S4 and S5, and Table S4).

**Figure 1:**
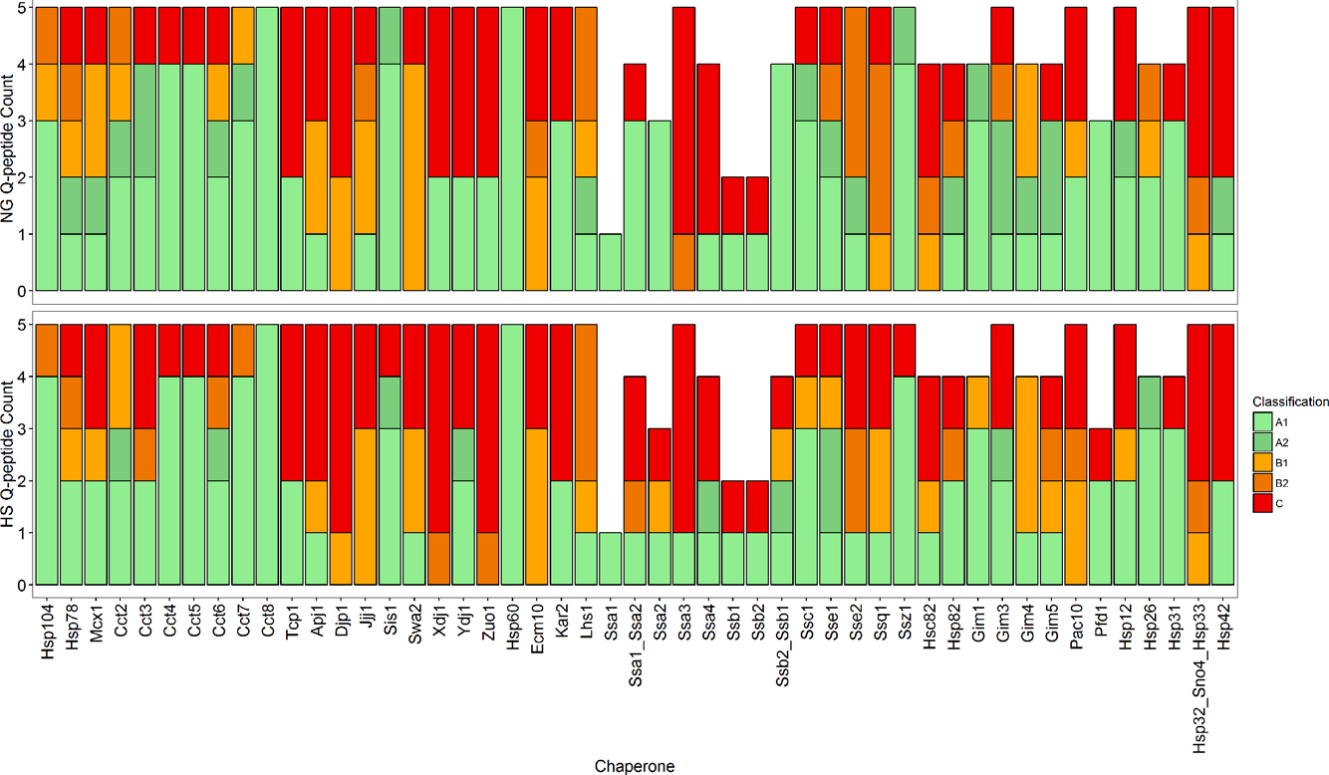
Classification of Q‐peptides on a per protein basis under normal growth (NG) and heat shock (HS) conditions. Q‐peptides were classified as ‘A1’ for those that were deemed suitable for absolute cpc value determination; ‘A2’ for those that had sub‐optimal features for robust quantification; ‘B1’ for those where the yeast analyte peptide was not above the limit of detection; ‘B2’ for those that did not pass the 1% FDR and ‘C’ for those peptides where neither the heavy ChapCAT or light yeast‐derived peptide ions were observed and so could not be used for quantification purposes. For particular HSP70 chaperone groups (*), these degenerate peptides were not used in the final quantification as unique peptides to each constituent chaperone were available. This was not the case for Hsp32_Sno4_Hsp33.

**Figure 2:**
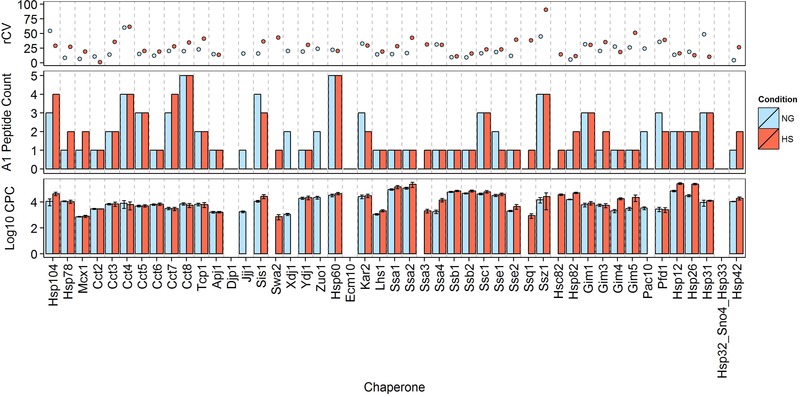
Absolute protein quantification performed using ‘A1’ Q‐peptides. cpc values are obtained for 36 chaperones under conditions of NG and HS. rCV values are below 40, with minor exceptions.

The majority of Q‐peptides, 88 and 84 peptides for NG and HS conditions, respectively, were classed as ‘A1’ and deemed suitable for protein quantification. Only 28 out of 116 peptides (24.1 %) were classed as ‘A2’ for NG samples and 14 out of 98 peptides (14.2 %) for the HS treated yeast (Fig. 1). Absolute protein abundances were determined by the median cpc across all biological replicate values for all ‘A1’ Q‐peptides targeting a particular chaperone. Under NG conditions, absolute protein levels were defined for 40 chaperone proteins, ranging from 700 to 114 000 cpc (Fig. 2). A slight increase in the median cpc level (from 7500 to 13 100 cpc) was observed under HS conditions, with values ranging from 700 cpc to 260 000 cpc (Fig. 2). Although 40 proteins were quantified under both growth conditions, there were changes in the proteins for which cpc values were not determined. Specifically, Pac10, Zuo1, Xdj1 and Jjj1 had ‘B1’ and ‘B2’ Q‐peptides in HS, but were quantified by ‘A1’ class peptides in NG. Similarly, Ssa3, Swa2, Ssq1 and Hsc82 had ‘B1’ and ‘B2’ Q‐peptides in NG but were quantified in HS. Only five chaperones from the 49 proteins targeted (Ecm10, Djp1 and those belonging to the protein group Hsp32_Sno4_Hsp33) failed to yield any absolute quantitative information. We estimate from the ‘B’ peptide data that Ecm10 and Djp1 lie below 2200 cpc in both conditions, whilst the combined abundance for Hsp32, Sno4 and Hsp33 is below 700 cpc in both conditions. Successful quantification was achieved for 36 proteins under both growth conditions (Fig. 2, Supporting Information Table S5).

It was predicted that increasing the number of Q‐peptides used for protein quantification could increase the confidence in the protein cpc value 20. We determined the rCV across biological replicates for all Q‐peptides on a per protein basis. For both NG and HS we observed that increasing the number of ‘A1’ Q‐peptides used for quantification generally leads, if anything, to a small increase in the median rCV; we did not observe a clear material gain in precision by attempting to increase the number of Q‐peptides (Supporting Information Fig. S6). Comparison of these chaperone cpc values with our previously reported values which used two Q‐peptides for quantification of *S. cerevisiae* grown under chemostat (steady‐state) conditions showed relatively good agreement: Spearman rank correlation coefficient for the log cpc values was 0.90 whilst the *R* ^2^ value for the linear regression was 0.795 (Supporting Information Fig. S7). The slight variation in cpc values are likely explained in large part by the differences in growth conditions; batch grown cultures encounter variable growth rate due to changing nutritional environment, whilst chemostat cultures remain steadily controlled. Changes in growth rates are known to affect protein (and/or transcript) levels, with proteins involved in the stress response reported to be up‐regulated under conditions of slow growth and carbon limitation, as is encountered in the previously analysed chemostat culture 37, 38. This is in agreement with our data where we observe a marginal increase in chemostat‐grown chaperone cpc values (as determined by a slope of 1.113). As an example, Hsp12 is calculated at 364 319 cpc and 68 598 cpc under chemostat and batch NG conditions, respectively, quantified by two Q‐peptides in both instances. Of these two Q‐peptides, LNDAVEYVSGR is used in both chemostat and batch datasets, yielding cpc values of 319 003 and 72 998 cpc, respectively, for the two datasets.

**Figure 3:**
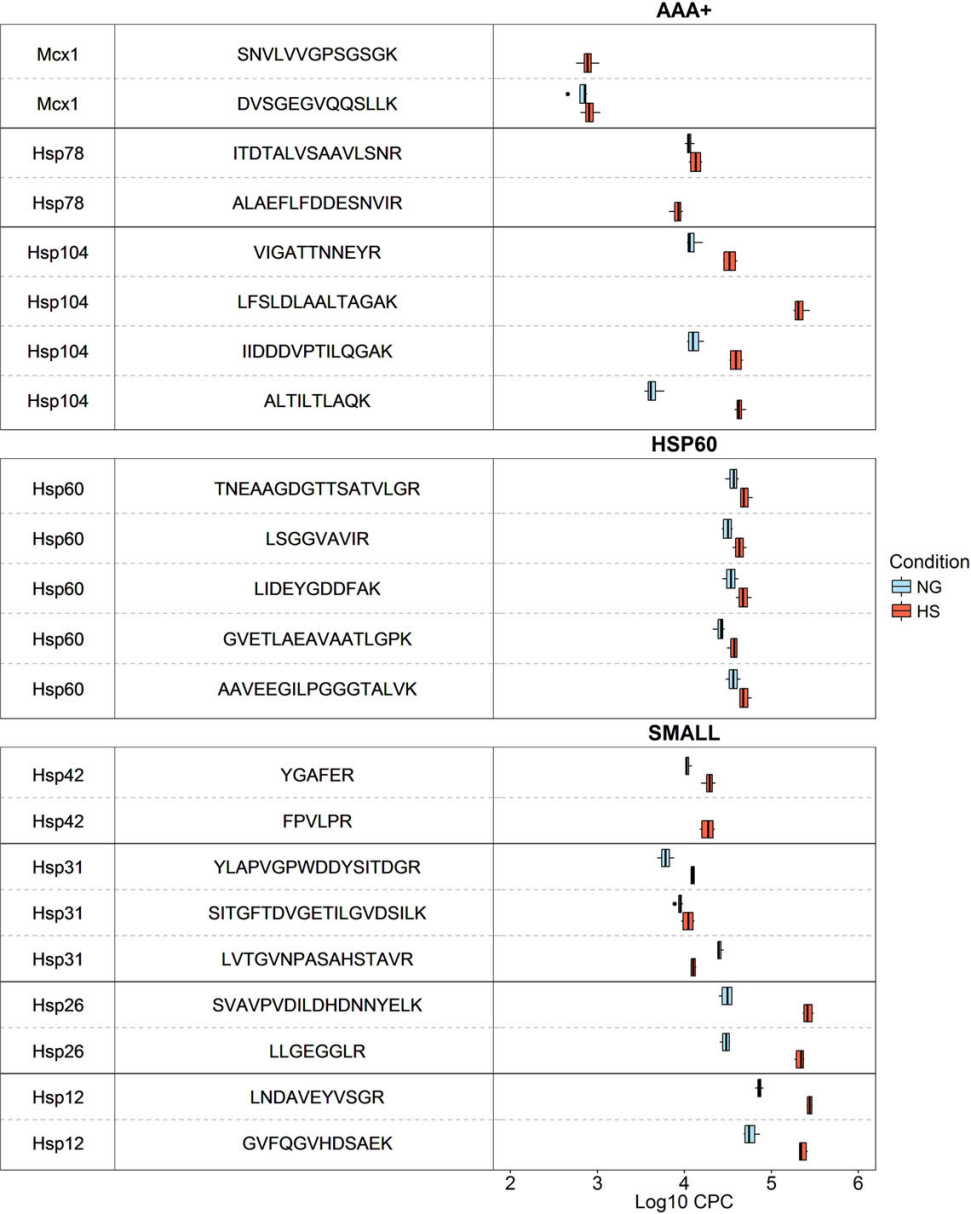
Observation of the spread of data points, reflecting rCV, for each ‘A1’ Q‐peptide for chaperones of the AAA+, SMALL and HSP60 classes. To investigate the cpc values on a per peptide basis and the effect on the target protein cpc value we observed the spread of biological replicate data points unique to each condition. We found that the rCV is not condition‐dependent.

Even with the amended Q‐peptide design considerations used here, the majority of proteins were quantified by two or fewer ‘A1’ class Q‐peptides. This illustrates one of the inherent challenges of peptide‐based targeted proteomics, namely that there are often few suitable quantotypic peptides for use in an absolute quantification experiment. We also noted an increased median rCV for the heat shock experiments: 28.0 compared to 18.9 under native growth conditions. To investigate, we examined the distribution of cpc values across biological replicates for Q‐peptides that were classed ‘A1’ in both NG and HS; a representative sample is shown in Fig. 3, the complete set in Supporting Information Figure S6. Although variation in measured cpc values increases marginally under HS, no clear systematic trend is observed. Rather, Fig. 3 highlights the good agreement between peptides common to a parent protein, with matched shifts in measured abundances observed between conditions (e.g. Hsp60 and Hsp26).

**Figure 4:**
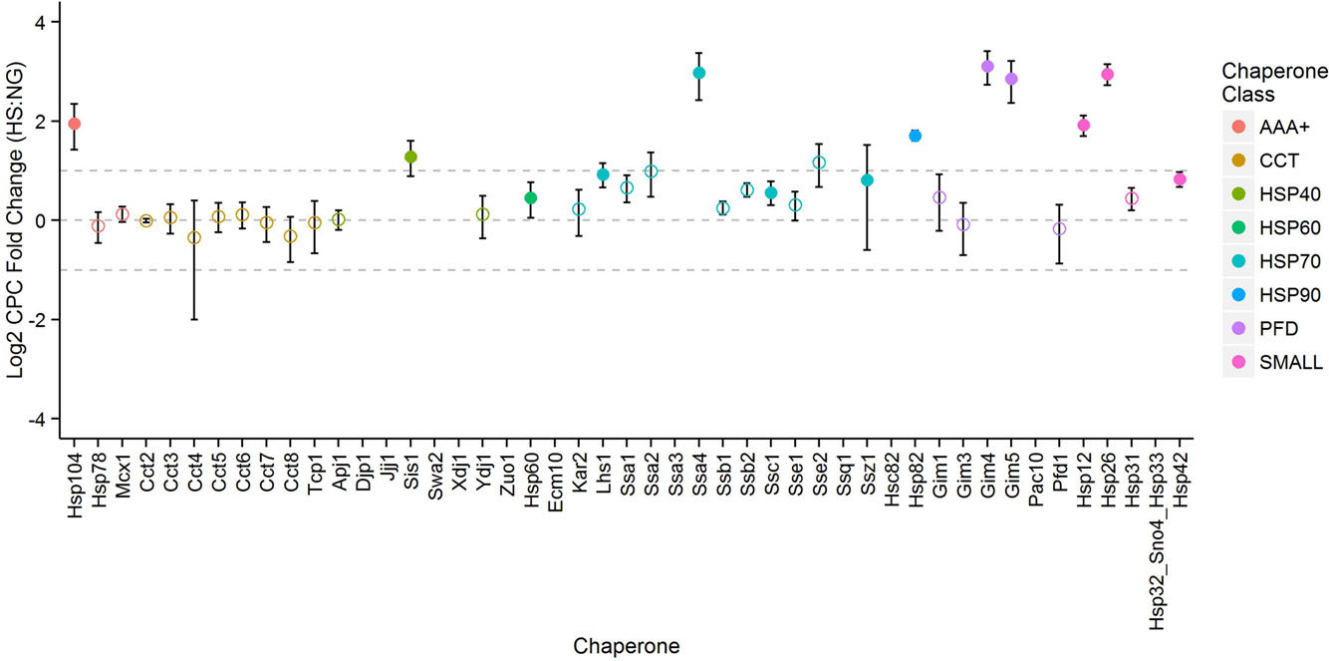
Upregulation of known Hsf1 targets. By performing an unpaired *t*‐test between all biological replicates used to determine final absolute protein abundance in normal growth and heat shock, the corresponding *p*‐values and thus significant changing proteins (filled points) were determined. Nine significantly upregulated chaperones are known direct gene targets of Hsf1 with roles in both the initiation and attenuation of the heat shock response. Our calculations of the errors for fold changes are explained in Supporting Information.

The ratio of cpc under NG and HS growth conditions was used to calculate the HSR‐induced fold change in each chaperone's abundance (Fig. 4). To calculate significance, an unpaired *t*‐test (*p*<0.05) was performed between all NG biological replicates and all HS biological replicates on a per protein basis for all ‘A1’ class Q‐peptides, correcting p‐values for multiple testing using the Benjamini–Hochberg FDR 39 approach (Supporting Information Table S5). For protein groups that shared non‐unique Q‐peptides, protein quantification was hypothesised to be the sum of the cpc values determined via unique Q‐peptides. For individual proteins within a group, cpc values were determined using unique Q‐peptides exclusively (see Supporting Information).

Thirteen proteins were observed to be significantly differentially expressed in response to heat shock (adjusted *p*‐value < 0.05), with a median fold change of 3.3; Hsp104, Sis1, Hsp60, Lhs1, Ssa4, Ssc1, Ssz1, Hsp82, Gim4, Gim5, Hsp12, Hsp26 and Hsp42 (Fig. 4). With the exception of Lhs1, Ssc1, Ssz1, Gim4 and Gim5, all of these significantly changing proteins are known direct gene targets of the HSR modulator, Hsf1 40. Indeed, the average fold‐change under HS for the 16 direct chaperone targets of Hsf1 (defined by Hahn et al. 40) quantified under both conditions was 1.9. These quantified Hsf1 targets fall into six chaperone subclasses: two AAA+ chaperones (Hsp78, Hsp104); three HSP40 chaperones (Apj1, Sis1, Ydj1); one HSP60 chaperone (Hsp60); six HSP70 chaperones (Kar2, Ssa1, Ssa2, Ssa4, Sse1 and Sse2); one HSP90 chaperone (Hsp82) and three small chaperones (Hsp12, Hsp26, Hsp42). Notably, no chaperones were found to be significantly downregulated in response to HS.

Cell viability depends on the ability to maintain proteostasis in response to heat shock. Failure to remove misfolded proteins via refolding mediated by chaperones may lead to their sequestration in designated protein quality control (PQC) foci or inclusion bodies to prevent cytotoxicity prior to their degradation. We observed significant upregulation of known chaperones in the HSR. Of the HSP70s, we found very low levels of Ssa4 (1800 cpc) in NG conditions, with significant upregulation of ∼seven‐fold (to 14 000 cpc) being observed following HS. The related heat‐inducible homolog Ssa3 was quantified only in HS (at 2000 cpc), with the native peptide being undetected in NG (considered as a class ‘B’ Q‐peptide), consistent with upregulation under HS. With regards to the HSP70 chaperones of the ER, we observed increased expression of Lhs1 (1.9‐fold, *p* = 0.02), whilst levels of Kar2, a direct target of Hsf1, were not significantly elevated. Ssa2, which has previously been reported to be non‐heat inducible 41, 42, was observed at 226 000 cpc under conditions of HS, almost two‐fold higher than the levels found under NG. However, issues of incomplete proteolysis and the necessary removal of some Q‐peptides as quantification standards meant that this apparent HS‐mediated change was deemed to be non‐significant (see Supporting Information for further discussion).

The HSP40 chaperone Sis1, a known co‐chaperone of the HSP70 chaperones Ssa1 and Ssa2, was significantly upregulated over two‐fold to 27 000 cpc. Whilst we observe Sis1 and Ydj1 to be the most abundant HSP40 chaperones (of those targeted) under NG conditions, their cpc values were much lower (11 000 and 19 000 cpc, respectively) in comparison to values reported in the literature determined via TAP‐tagging and quantitative western blotting approaches (20 300 and 119 000 cpc, respectively), albeit for a different yeast strain (BY4741) 17. Sis1 is able to stimulate the ATPase activity of HSP70 chaperones, shuttling substrates between the cytosol and nucleus. It has also been linked with targeting of misfolded substrates to the PQC‐degradation system and a role in protection from prion toxicity alongside Hsp104 43, 44, 45, 46. In agreement with previous findings, the AAA+ class chaperone Hsp104 was low under NG conditions (11 000 cpc) but increased significantly (four‐fold to 42 000 cpc, *p* = 8.94×10^−7^) after exposure to HS. Hsp104 functions in a complementary role to the water‐displacing molecule trehalose, stabilising proteins at physiological concentrations 5, 47. Hsp104 is able to cooperate with Ydj1 (HSP40) and Ssa1 (HSP70) to refold previously denatured proteins that have become aggregated 48. Unlike conventional chaperones, Hsp104 functions specifically to dissociate aggregates that have formed due to overloaded cellular chaperone capacity, freely localising to, and removing those proteins that are terminally misfolded and contained within the perivacuolar insoluble protein deposit and juxtanuclear compartments (the PQC foci) 5, 48.

Literature suggests marked down‐regulation of the HSP70 class ribosome‐associated chaperones Ssb1 and Ssb2 in response to thermal stress, inferred from mRNA abundances, albeit at prolonged times and varying temperatures 49, 50. However, at the protein level, we did not observe a significant difference; both Ssb1 and Ssb2 were present in HS at 69 000 cpc, compared to 58 000 and 49 000 cpc in NG for Ssb1 and Ssb2, respectively. Given that the correlation observed between mRNA levels and protein abundances is generally considered to be modest 51, particularly under transitions associated with stress 52, our results suggest post‐transcriptional regulation is in play. mRNA half‐lives are typically shorter than those of proteins, so a decrease in mRNA abundance may not be reflected immediately at the protein level. Conceivably, a reduction in Ssb1 and Ssb2 protein levels might be observed upon prolonged heat shock conditions; it would be interesting to compare the absolute cpc numbers of chaperones in response to heat shock across various *S. cerevisiae* strains over extended time periods, however this was not the focus of our current research.

Of the HSP90 family, Hsp82, which functions in the final steps of protein folding and complex assembly receiving client proteins from the HSP70 chaperones via the co‐chaperone Sti1 5, 13, was found to be significantly upregulated three‐fold to 50 000 cpc. Similarly, Hsc82 was also elevated in HS, being quantified only under these conditions (akin to Ssa3), at 36 000 cpc. These data support previous studies that reported modest increases in Hsc82 levels in response to heat shock compared to larger Hsp82 changes 53. Although Hsp82 is thought to work with Ssa1/2 to deactivate Hsf1 expression following cellular recovery 54, there was no significant heat stress‐induced change in these co‐regulators, in contrast to our observations for Hsp82.

Interestingly, Gim4 and Gim5 which are members of the prefoldin (PFD) class of chaperones, were both observed to be significantly unregulated by 8.6‐fold and 7.2‐fold, respectively, following HS; neither of these are reported to be direct targets of Hsf1 40. However, although levels of both Gim4 and Gim5 were HS‐induced, there was no significant increase in the levels of the remaining four subunits of the heterohexameric PFD chaperone complex.

Three of the four ‘small’ class chaperones quantified under both conditions (Hsp12, Hsp26 and Hsp42) were observed to be significantly upregulated. Hsp26 exhibited the greatest significant change following HS, with its absolute abundance increasing to 235 000 cpc, almost an eight‐fold increase over that under NG conditions. Although significant, the ∼two‐fold upregulation of Hsp42 was notably lower than that of Hsp12 and Hsp26, whose levels increased 3.8‐fold and 7.7‐fold in response to HS, respectively. These observations are consistent with previous studies which suggest that most small HSP family members, apart from Hsp42, are functionally inactive under NG conditions is 55. Small class chaperones are known to form large oligomeric complexes containing unfolded protein within their hollow structures, thus protecting from aggregation until refolding can occur 5, 56, 57.

### 3.3 SRM‐corrected label‐free quantification

We matched the absolute quantification of the chaperones with a label‐free study of the whole proteome using the same NG and HS samples, quantifying 1671 and 1816 proteins, respectively, with 1644 yeast proteins in common between the two conditions (with a maximum Q‐value for protein identification of 0.0091). In this set, 37 chaperone proteins were also quantified in a relative manner, one of which, interestingly, was Sec63, a protein which was not targeted in our SRM experiments as it was included in ChapCAT010 which failed to express at sufficient levels (Supporting Information Table S1).

**Figure 5:**
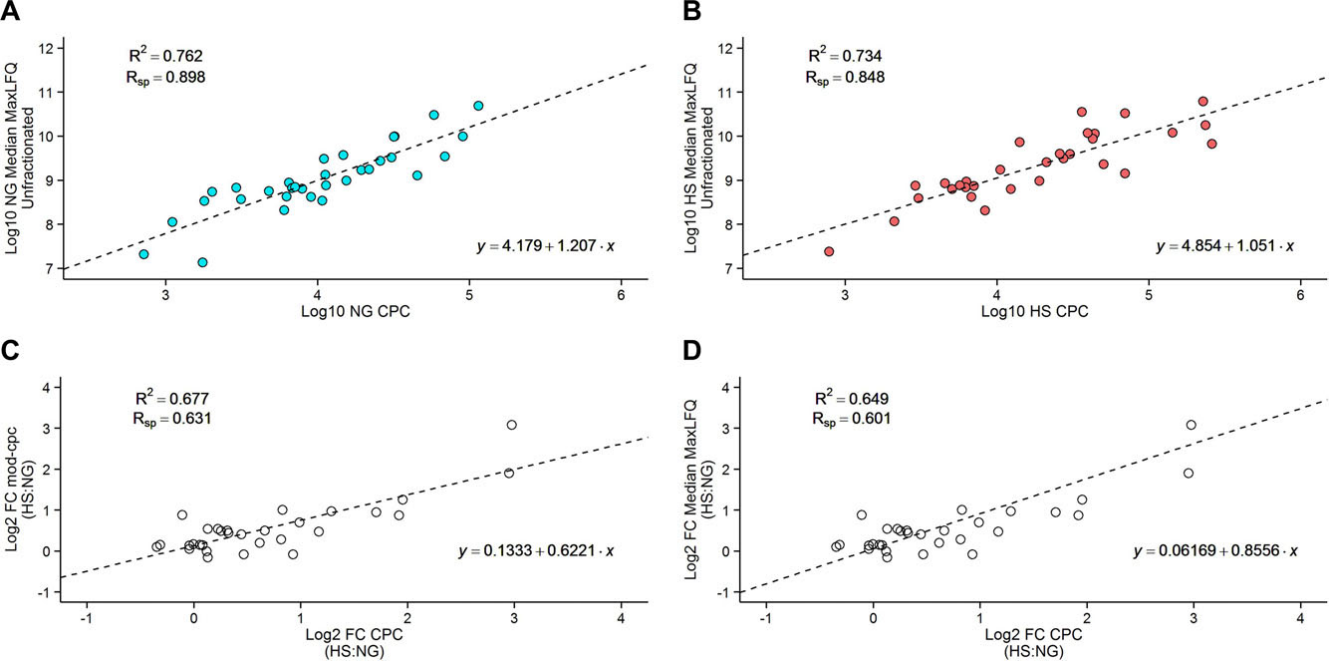
Assessment of the abilities of relative quantification and MaxLFQ SRM‐normalisation. After performing an unfractionated label free experiment we compared the relative quantification of chaperones observed in NG conditions (A) and HS conditions (B). We performed MaxLFQ SRM‐normalisation to obtain mod‐cpc values for chaperones, and determined their fold changes according to their mod‐cpc values. (C) Upon comparison of these fold changes to absolute fold changes, we observed decreased agreement as a magnitude of the error in the model. (D) We assessed the ability of relative quantification to accurately define fold errors, and found that the agreement between the relative fold errors and the absolute fold errors is less than the agreement between the fold errors obtained following MaxLFQ SRM‐normalisation.

Protein abundance determined using label‐free quantification displayed good agreement with respect to SRM‐based QconCAT quantification, comparing the chaperone cpc values with their corresponding median MaxLFQ intensities reported by MaxQuant. A logged comparison of the 32 chaperones identified under NG conditions (comparison set A) produced a Spearman rank correlation coefficient of 0.898, with an *R* ^2^ value of 0.762 (Fig. 5A). For the 31 chaperones in common in HS (comparison set B) the Spearman rank correlation coefficient was 0.848, whilst the *R* ^2^ value for the linear regression was 0.734 (Fig. 5B).

To convert the label‐free quantification data to absolute values, we performed condition‐dependent linear regression using the R package ‘aLFQ’ 23 to predict cpc values from the median MaxLFQ intensities modelled on the cpc values for chaperones identified in both the SRM and unfractionated label‐free datasets. Using a leave‐one‐out cross‐validation, this yielded a mean fold error of 1.8 for comparison set A and 2.0 for comparison set B. This regression approach normalised the label‐free MaxLFQ values to compute SRM‐corrected label free values (mod‐cpc) for all 1644 proteins that were identified under both NG (*R* ^2^ = 0.762; Slope = 1.21; Intercept = 4.18; F = 96.16; *p* = 7.21 × 10^−11^) and HS (*R* ^2^ = 0.734; Slope = 1.05; Intercept = 4.85; F = 79.92; *p* = 7.87 × 10^−10^). A similar approach towards absolute quantification of the proteome has been performed in *E. coli* by Schmidt and colleagues 58.

To determine the validity of SRM normalisation of the label‐free quantification data in this manner, we compared the mod‐cpc fold change in 30 ChapCAT‐quantified proteins in response to HS. As mod‐cpc under either NG or HS had a mean fold error of two, we anticipated a greater error when calculating fold changes (Fig. 5C). This proved to be the case, with a lower but still reasonable Spearman rank correlation coefficient of 0.631 and an *R* ^2^ of 0.677. Finally, we assessed the ability of unfractionated label free experiments to observe significant differences in chaperone protein abundance between NG and HS. To do so, we compared the fold change of median MaxLFQ (HS / NG) for 30 chaperones identified in both conditions to their cpc fold change counterparts. This produced a result with *R* ^2^ of 0.649 (Spearman's rank correlation coefficient was 0.601), again showing reasonable agreement but slightly worse than the mod‐cpc versus cpc acquired fold changes (Fig. 5D). The aLFQ‐based normalisation was, therefore, slightly superior in estimating protein fold changes than those determined by a standard label free experiment. When comparing the MaxLFQ fold‐changes and mod‐cpc fold‐changes using an unpaired Wilcoxon test (U test, *p*<0.05), we observed no significant difference in the ranks of the fold changes of the chaperones (*p* = 0.93). We also assessed whether significantly upregulated chaperones in the SRM‐cpc dataset were present in the top ten equivalent set in the mod‐cpc and MaxLFQ datasets, with both mod‐cpc and MaxLFQ datasets ordered by decreasing fold changes (Supporting Information Tables S7 and S8). We observed seven significantly upregulated chaperones (according to our SRM dataset) in both the top ten for the mod‐cpc and MaxLFQ datasets. However, in the MaxLFQ dataset, we observed only three significantly upregulated chaperones according to their adjusted *p*‐value, determined via an unpaired *t*‐test across the NG and HS biological replicate values for MaxLFQ intensity. Due to our modelling strategy we cannot determine *p*‐values for the mod‐cpc dataset. However, according to the top ten approach, using MaxLFQ SRM‐normalisation improved our chances of identifying significant changes as determined by the gold standard SRM method compared to using a purely label‐free approach based on MaxLFQ intensities.

According to our MaxLFQ SRM‐normalised model for 1644 proteins in NG and HS conditions (Supporting Information Table S9), the median cpc under NG and HS was 3700 and 3500, respectively, with a median fold change of 0.97. Performing an unpaired Wilcoxon test (U test, *p* < 0.05) on protein concentration across biological replicates determined via a Bradford Assay indicates no significant change in response to HS (*p* = 0.80). No change in cell size was observed during cell counting, with the average size of a cell under NG and HS (4.18 μm). Total protein abundance of the 1644 proteins increased in response to HS to 18 482 888 mod‐cpc, a 1.18‐fold change. However, on a local scale we observe few proteins with significant differences that might contribute to this total abundance change. Due to the nature of our modelling approach we define significance as a fold change +/– 2‐fold. We identify five proteins (Hsp26, Ssa4, Hsp104, Rgi1 and Hsp42) with a fold change over 2.0 in response to heat shock, whilst only Elo2 becomes significantly downregulated under the same conditions, exhibiting a fold change of less than 0.5. In addition to the previously identified Hsp26, Hsp42, Hsp104 and Ssa4, Rgi1 (Respiratory Growth Induced protein 1) also becomes upregulated (2.2‐fold) upon HS. Although the precise function of Rgi1 has yet to be elucidated, it appears to have a role in regulating energy metabolism and drug resistance, with high expression levels reported under a wide range of conditions, inclusive of high temperatures, cold stress and the unfolded protein response 59, 60, 61. In contrast, Elo2, a fatty acid elongase localised to the endoplasmic reticulum, becomes downregulated 0.5‐fold. Elo2 is involved in sphingolipid biosynthesis (essential components of membranes and thus important for cellular integrity) and transport from the late endosome to the vacuole as part of the secretory pathway 62. Its paralog, Elo1, was not identified in the label‐free analysis.

The observation that the proteome does not exhibit significant changes globally is not surprising. During the HSR, the cell would attempt to maintain homeostasis such that the global protein abundance would remain constant. As such, one would expect very few significant changes. However, this mod‐cpc analysis does highlight a limitation with normalisation in large scale proteomics; normalising both NG and HS median MaxLFQ intensities may result in underrepresentation of true significant changes that are occurring. Here, we are able to identify only those that are on the extreme ends of regulation. Despite this, we are able to provide protein‐level evidence, in terms of absolute copies per cell quantification, of the yeast cell's ability to maintain overall proteostasis and the ability to adapt to heat shock conditions.

## 4 Concluding remarks

Targeting known chaperones in *S. cerevisiae* with more than two Q‐peptides in the design of a QconCAT standard construct has added to the existing pool of available Q‐peptides for quantification of chaperone proteins from *S. cerevisiae*, and increased the total number of proteins quantified. Of the 49 chaperones targeted, 36 proteins were quantified in absolute terms, defining cpc values under both standard growth conditions and following exposure to HS. We observe a significant increase in protein levels for chaperones known to participate in the HSR, with eight of the chaperones identified as significantly upregulated being direct gene targets of Hsf1. Absolute levels of these Hsf1‐targeted chaperones increased over two‐fold in response to HS. It is widely accepted that chaperones and their co‐chaperones liaise, particularly when challenged by abundant misfolded protein, and we were able quantify chaperones known to cooperate in this manner. As expected, chaperones of the small class, which bind misfolded proteins to prevent aggregation, were all upregulated. Of note, Hsp26, which has known roles in the protein disaggregation chaperone pathway, was significantly upregulated eight‐fold. Comparison of the NG cpc values for those chaperones also quantified under steady‐state (chemostat) growth conditions in our previous QconCAT‐based study 20 shows relatively good correlation. The small differences observed could be explained by the differences in growth conditions and the additional Q‐peptides used for quantification.

We were able to extend this absolute quantification of chaperone subset of proteins to 1644 via unfractionated label‐free experiments in NG and HS conditions. By performing median MaxLFQ SRM‐normalisation, we were able to model cpc values to within two‐fold of the actual cpc abundance, thus providing 1644 cpc values for proteins under both NG and HS conditions. The complete ChapCAT designs, validated transitions and digestion time course experiments have been deposited for public use in the PASSEL database (accession PASS00781). The wealth of quantitative data enables us to work towards understanding the chaperone‐client ‘interactome’ in response to HS, and will provide important insights into the cellular protection and recovery from HS.

*The authors declare no conflict of interest*.

## Supporting information

As a service to our authors and readers, this journal provides supporting information supplied by the authors. Such materials are peer reviewed and may be re‐organized for online delivery, but are not copy‐edited or typeset. Technical support issues arising from supporting information (other than missing files) should be addressed to the authors.

Figure S1) Extraction of ChapCAT from inclusion bodies. Expressed ChapCAT (shown are ChapCAT001 and ChapCAT003) was isolated from the inclusion bodies of E. coli and subject to further purification. Validation experiments were performed by western blotting with an α‐His antibody. SM: starting material; SF: soluble fraction; IB: inclusion bodies. ChapCATs are indicated with an arrow.

Table S1) Expression Protocol for ChapCATs in minimal medium
Following expression testing in LB medium, the optimum expression conditions were determined. For ChapCATs that encountered difficult expression in LB medium, the addition of 10 mM benzyl alcohol was used to attempt higher levels of quantification. This was not successful for the ChapCAT010 construct and so was discontinued after initial testing.
