# Supplementary material for: Absolute protein quantification of the yeast chaperome under conditions of heat shock
Source: Proteomics. 2016 Jul 22;16(15-16):2128–40. doi: 10.1002/pmic.201500503 (PMC4996341; doi:10.1002/pmic.201500503)
Supplement: Supplementary file 1 — Figure S1) Extraction of ChapCAT from inclusion bodies. Expressed ChapCAT (shown are ChapCAT001 and ChapCAT003) was isolated from the inclusion bodies of E. coli and subject to further purification. Validation experiments were performed by western blotting with an α‐His antibody. SM: starting material; SF: soluble fraction; IB: inclusion bodies. ChapCATs are indicated with an arrow. [file PMIC-16-2128-s001.docx]

**Supplementary Information**

***QconCAT-driven absolute protein quantification of the yeast chaperome under conditions of heat shock***

Rebecca J. Mackenzie, Craig Lawless, Stephen W. Holman, Karin Lanthaler, Robert J. Beynon, Chris M. Grant, Simon J. Hubbard & Claire E. Eyers

[Supplementary Methods 2](#_Toc450052735)

[1. Expression testing of ChapCAT 2](#_Toc450052736)

[2. Further details of mass spectrometry 2](#_Toc450052737)

[3. *In silico* retention time window adjustment 3](#_Toc450052738)

[Supplementary Results & Discussion 4](#_Toc450052739)

[1. Absolute quantification via non-unique Q-peptides 4](#_Toc450052740)

[2. Digestion Time Course Experiment 5](#_Toc450052741)

[3. Classification of ‘A1’ and ‘A2’ Q-peptides 5](#_Toc450052742)

[4. The absolute cpc levels of Ssa2 under NG and HS 7](#_Toc450052743)

[5. Calculation of error bars for fold change between NG and HS 7](#_Toc450052744)

[Figure S1) Extraction of ChapCAT from inclusion bodies. 7](file:///C:\Users\becki\Dropbox\Rebecca_Mackenzie_Proteomics_Paper\Response_to_Reviewers\Filesmodified_(Forsubmission)\Final_to_submit\Supplementary_info_v5.2.docx#_Toc450052745)

[Figure S2) Purification of the ChapCAT protein from the *E. coli* inclusion bodies. 8](file:///C:\Users\becki\Dropbox\Rebecca_Mackenzie_Proteomics_Paper\Response_to_Reviewers\Filesmodified_(Forsubmission)\Final_to_submit\Supplementary_info_v5.2.docx#_Toc450052746)

[Figure S.3) Digestion Time Course 9](file:///C:\Users\becki\Dropbox\Rebecca_Mackenzie_Proteomics_Paper\Response_to_Reviewers\Filesmodified_(Forsubmission)\Final_to_submit\Supplementary_info_v5.2.docx#_Toc450052747)

[Figure S4) Decision tree for classification of A peptides for suitability for quantification. 10](file:///C:\Users\becki\Dropbox\Rebecca_Mackenzie_Proteomics_Paper\Response_to_Reviewers\Filesmodified_(Forsubmission)\Final_to_submit\Supplementary_info_v5.2.docx#_Toc450052748)

[Figure S5) Classification of an ‘A’ peptide to ‘A2’. 11](file:///C:\Users\becki\Dropbox\Rebecca_Mackenzie_Proteomics_Paper\Response_to_Reviewers\Filesmodified_(Forsubmission)\Final_to_submit\Supplementary_info_v5.2.docx#_Toc450052749)

[Figure S6) Observation of the spread of data points, reflecting rCV, for each ‘A1’ Q-peptide for every chaperone. 12](file:///C:\Users\becki\Dropbox\Rebecca_Mackenzie_Proteomics_Paper\Response_to_Reviewers\Filesmodified_(Forsubmission)\Final_to_submit\Supplementary_info_v5.2.docx#_Toc450052750)

[Figure S7) Comparison of protein CPC values for 34 common yeast chaperones from this study (ChapCAT) and our previously published study by Brownridge et al., (CopyCAT). 13](#_Toc450052751)

[Figure S8) Absolute quantification of chaperones in NG and HS conditions. 14](file:///C:\Users\becki\Dropbox\Rebecca_Mackenzie_Proteomics_Paper\Response_to_Reviewers\Filesmodified_(Forsubmission)\Final_to_submit\Supplementary_info_v5.2.docx#_Toc450052752)

[Table S1) Expression Protocol for ChapCATs in minimal medium 15](#_Toc450052753)

[Table S2) Transition list for top 3 SRM ChapCAT Experiment 15](#_Toc450052754)

[Table S3) Quantification on a per peptide basis 15](#_Toc450052755)

[Table S4) Digestion Time Course rate constants and half-lives 15](#_Toc450052756)

[Table S5) Absolute quantification of chaperones in NG and HS conditions 15](#_Toc450052757)

[Table S6) Label free single shot protein identifications by MaxQuant 15](#_Toc450052758)

[Table S7) Comparing top 10 significant protein fold changes between cpc and mod-cpc 16](#_Toc450052759)

[Table S8) Comparing top 10 significant protein fold changes between cpc and MaxLFQ 16](#_Toc450052760)

[Table S9) MaxLFQ SRM-Normalised cpc values for 1644 proteins in NG and HS 16](#_Toc450052761)

## Supplementary Methods

### 1. Expression testing of ChapCAT

During initial protein expression testing in LB media, ChapCAT002, ChapCAT006 and ChapCAT010 failed to generate detectable protein following induction with 1 mM Isopropyl β-D-1-thiogalactopyranoside (IPTG). The constituent Q-peptides for these three ChapCAT constructs were thus subjected to a random peptide reshuffle and the ChapCAT construct resynthesised (PolyQuant GmbH, Germany). The re-shuffled ChapCAT002 and ChapCAT006 constructs subsequently expressed protein. However, the new ChapCAT010 still failed to yield detectable protein levels and the decision was made not re-shuffle and re-synthesise this standard. Instead, attempts were made to increase expression levels by varying the growth time prior to protein induction, addition of benzyl alcohol, varying IPTG concentration for induction and the incubation time following induction for all ChapCATs displaying low levels of expression (ChapCAT002 and ChapCAT009) (Supplementary Information Table 1). For ChapCAT009, the incubation time was reduced to 3 hours from 5 hours in the likelihood that the longer incubation time may have allowed for ChapCAT degradation.

For expression and purification of ChapCATs in minimal media with heavy labelled amino acids, the protocol differed from published literature as 10 mg/ml of each individual amino acid was incorporated into the media. Upon successful expression in light media, ChapCAT constructs were expressed and purified in heavy medium (Figure S.1 & S.2).

### 2. Further details of mass spectrometry

Each tryptic digest was analysed by LC-MS using a nanoAcquity UPLC^TM^ system (Waters, Manchester) coupled to a Synapt^TM^ G2-Si mass spectrometer (Waters, Manchester) to check complete digestion and to quantify the ChapCAT. One µl of sample (corresponding to approximately the protein equivalent of 100,000 cells) was loaded onto the trapping column (Symmetry C18, 5µm packing material, 180 µm x 20 mm, Waters, Manchester) using partial loop injection, for 3 minutes at a flow rate of 5 µL min^-1^ with 99.9 % A (0.1 % formic acid) : 0.1 % B (99.9 % ACN, 0.1 % formic acid). The sample was resolved on an analytical column (nanoAcquity UPLC^©^ HSS T3 C18 75 µm x 150 mm 1.7 µm column, Waters, Manchester), using a gradient of 97 % A 3 % B to 60 % A 40 % B over 60 minutes; then washed 60% A 40% : B before re-equilibrating to starting conditions. Data was acquired by the mass spectrometer in a data independent program with a collision energy ramp of 15 to 40 eV for increased energy scans.

SRM analysis was performed using a nanoAcquity UPLC^TM^ system coupled to a Xevo^TM^ TQ(-S) triple quadrupole mass spectrometer (Waters, Manchester). The mass spectrometer was operated in scheduled SRM mode with Q1 and Q3 operating at unit resolution. The program was set to acquire 15 data points over a 30 s chromatographic peak within a 3 minute window. The final transition list was divided to achieve a minimum dwell time of 50 ms and each sample analysed with all subsequent transition lists.

The data-dependent label free analysis was performed using a Dionex Ultimate 3000 RSLC^TM^ system (ThermoScientific, Hemel Hempstead) coupled to a Q-Exactive HF mass spectrometer. Sample equivalent to 1 µg of protein concentration was loaded onto the trapping column (ThermoScientific PepMap® 100 C18, 300 µm X 5 mm) using partial loop injection, for 7 minutes at a flow rate of 9 µL min^-1^ with 97.9:2:0.1% (v/v) H_2_O:MeCN:TFA. The sample was resolved on an analytical column (Easy-Spray C18 75 µm X 500 mm, 2 µm column) using a gradient of 96.2 % A (0.1 % formic acid) 3.8 % B (79.9 % MeCN, 20 % H_2_O, 0.1 % formic acid) to 50 % A 50 % B over 90 minutes; then washed at 1% A 99% B before re-equilibrating to starting conditions at a flow rate of 300 nL min^-1^.

### 3. *In silico* retention time window adjustment

For every ‘A’ peptide (prior to assignment to ‘A1’ or ‘A2’ class) we observed the transition profiles in Skyline [28]. Skyline automatically assigns a peak to each Q-peptide based on the co-elution of its transitions. We confirmed this assignment for every peptide for each sample visually. The default ‘Peak Boundaries’ report was exported from Skyline (File > Export > Report > Peak Boundaries) and peak widths were calculated by comparing the minimum start time and maximum end time for each transition for each peptide (heavy and light). A file containing all peak widths for all Q-peptides was concatenated and the distribution of peak widths was observed. We found that all ‘A’ Q-peptides eluted within a one minute window, and used this to define our new *in silico* retention time window to aid mProphet peak group selection. An in-house Perl script read in the concatenated ‘Peak Boundaries’ file, defined a one minute retention time window (+/- 30 seconds) about the median of the minimum start time and the minimum end time for each peptide. Subsequently, for each peptide, the transition groups listed in the processed mrml.xml files were adjusted to only include *m/z* values within the new retention time window. The adjusted mrml.xml files were then processed with mQuest and mProphet as previously described [5]. mQuest search parameters were contained within the file ‘copy.params’.

copy. params

use_reference 1

allow_pgpair_wo_reference_partner 0

allow_pgpair_wo_target_partner 1

make_dummy_peakgroup 1

fill_pgs_with_noise 1

light_label light

heavy_label heavy

reference_isoform heavy

minSN_target 5

minSN_reference 5

select_nbest_peakgroups_target 4

select_nbest_peakgroups_reference 2

use_decoy 1

decoy_schema AQUA

main_vars log10_total_xic|intensity_correlation_with_assay|xcorr_coelution_score|xcorr_shape_score|light_heavy_correlation|light_heavy_shape_score|light_heavy_coelution_score|abs_Tr_deviation

main_score light_heavy_shape_score

min_peak_width 7

select_nbest_peaks 5

max_Tr_difference 5

denoise_parameter1 5

## Supplementary Results & Discussion

### 1. Absolute quantification via non-unique Q-peptides

The chaperone protein pairs Ssa1, Ssa2 and Ssb1, Ssb2 were targeted by Q-peptides that were both unique to the protein, and unique to the protein pair. As such, it was anticipated that the abundance of the protein pair would be the sum of the unique protein abundances, and therefore could be used alongside unique Q-peptides to determine chaperone abundance. For the protein pair Ssa1_Ssa2, the NG absolute abundance was 125,000 copies per cell (cpc), whilst unique protein abundances were 90,000 cpc (determined via a single unique Q-peptide) and 78,000 cpc (determined via 3 unique Q-peptides) for Ssa1 and Ssa2 respectively. The sum of the unique protein abundances was higher than the combined non-unique protein pair abundance and as such, protein cpc values for these proteins were determined using unique Q-peptides only for both NG and HS. For the protein pair Ssb1_Ssb2, NG absolute abundance was 84,000 cpc, whilst unique protein abundances were 45,000 cpc and 58,000 cpc for Ssb2 and Ssb1 respectively. Again, the sum of the unique protein abundances was higher than the combined non-unique protein pair abundance, and as such the NG and HS absolute quantifications for these proteins were performed using unique Q-peptides only.

### 2. Digestion Time Course Experiment

To determine the efficiency of digestion, 10 μL samples were taken at time intervals (0 min, 1 min, 2 min, 5 min, 10 min, 20 min, 50 min, 120 min240 min, 270 min and 1230 min) and digestion halted via addition of 10 μL of 5% (v/v) TFA. Samples were subjected to SRM analysis as described in the manuscript. Raw files were subsequently loaded into Skyline and a report generated containing the respective heavy (QconCAT) and light (yeast analyte) peak areas for peptides targeted. Each peak area was transformed to a percentage by dividing by the maximum peak area for the label type for the particular peptide. In order to follow the digestion reaction, a pseudo-first order equation was defined (Equation 1).

Equation 1)

$$P_{t}=100-\left( \left( 100 - P_{t0} \right)* e^{\left( -k*t \right)} \right)$$

Where *P* is the percentage of peptide at time *t* and *k* is the rate constant to be determined. Using a non-linear modelling (nls) package in the statistical software package R, a value of *k* was determined for a peptide with respect to both heavy and light counterparts. The digestion half-life of a peptide is determined by Equation 2.

Equation 2)

$$t_{\frac{1}{2}}= \frac{\ln\left( 2 \right)}{k}$$

Digestion was deemed complete within 5 half-lives. We were therefore able to determine the degree to which a peptide had completed digestion (Figure S.3). If a peptide had not completely digested in either the standard or analyte to a similar degree, it was deemed likely to result in erroneous quantification and therefore not included in the cpc calculation.

### 3. Classification of ‘A1’ and ‘A2’ Q-peptides

For each Q-peptide observed in both heavy ChapCAT and the light yeast sample (‘A’ peptide), it was further analysed to determine suitability of the Q-peptide for absolute quantification (Figure S.4). Firstly, the rCV was calculated across the biological replicates. Peptides with an rCV over 30 were flagged as potential ‘A2’ peptides. The flagged peptides were then investigated in terms of the following XIC properties: the fragment ion transition profile between heavy and light and selection of the correct peak group by mProphet (aided with the *in silico* retention time reducing program). Peptides that had suspect transition profiles had the offending transitions removed in extreme instances and were reprocessed using the described mProphet pipeline. If this did not improve rCV and peak group selection, such peptides were classed as ‘A2’. Each peptide was searched against the experimentally verified post-translational modification (PTM) site dataset available at dbPTM (<http://dbptm.mbc.nctu.edu.tw/>), first searched in September 2012 for ChapCAT design, again searched in July 2015 during SRM processing to update any PTM matches. If an ‘A’ peptide was reported to have a PTM, it was classified an ‘A2’ and not used for quantification.

We reasoned that peptides from the same protein would have similar directional abundance changes between NG and HS, in that we expected all peptides targeting the same protein to increase/decrease in abundance to reflect the protein fold change. If a single peptide did not follow the same pattern outlined by all other peptides targeting the same protein we elected it to be an ‘A2’ peptide, as it likely contains sub-optimal features that hindered the accuracy of protein quantification. In the same reasoning, if a protein was targeted by > 2 ‘A’ Q-peptides, with a single Q-peptide having a two-fold higher/lower abundance than the median cpc of the remaining peptides, we investigated further into the peptide rCV, transition profiles and mProphet peak group selection, and if reasons identified, it was classified an ‘A2’ peptide.

One such example is the peptide TQIDIQLKPGWK targeting Sis1 (Figure S.5). Under NG conditions, the peptide had a low cpc (9800 cpc) compared to other peptides from the same protein, but is not outside two-fold of the median cpc across ‘A’ peptides in NG (12,000 cpc) and had a low rCV of 8.76. However, under HS conditions, the cpc value for TQIDIQLKPGWK was almost four-fold that obtained for the other Q-peptides targeting the same protein thus outside of the 2-fold median cpc for HS (32,000 cpc), with the largest rCV (54) in HS for Sis1. Upon investigation of transition profiles for TQIDIQLKPGWK in HS in Skyline [28], we found that even at 10 fmol ChapCAT, the ChapCAT signal was less than that of the analyte peptide and observed large fluctuations in yeast signal intensity between biological replicates. In HS2 we found excessive tailing in the chromatography of the peak, however all biological replicates were able to pass a 1 % FDR via mProphet processing. Together with the high rCV, chromatographic sampling issue and having a value outside two-fold of the median cpc value for HS we determined this peptide to be an outlier, thus was classified an ‘A2’ peptide.

In instances where there were only two ‘A’ peptides with more than two-fold difference in absolute cpc value, the Q-peptide with the highest cpc value was taken as the ‘A1’ peptide for quantification to account for the fact that a loss of signal is observed if the native peptide is potentially post-translationally modified. This was in accordance with the methodology described by Brownridge and colleagues [5]. One example of this was with the ‘A’ class Q-peptide SNVLVVGPSGSGK (Mcx1) which in NG conditions had a value of 320 cpc, and an rCV across biological replicates of 30. The only other available ‘A’ class Q-peptide for Mcx1 in NG conditions was DVSGEGVQQSLLK with a value of 714 cpc and an rCV of 6.6. Here, the latter was classed as the ‘A1’ Q-peptide and therefore used for protein quantification, whilst the former was classed an ‘A2’ Q-peptide.

### 4. The absolute cpc levels of Ssa2 under NG and HS

We found the previously considered non-heat inducible Ssa2 present at 226,000 cpc HS at almost 2-fold higher abundance than its homolog Ssa1. Although there are 3 ‘A’ class peptides for Ssa2, the endogenous protein was not digested to completion: the yield of the light peptides, EEFDDQLK and ELQEVANPIMSK, was only 72% and 82% respectively. TTPSFVGFTDTER was thus the only ‘A1’ Q-peptide that could be used for quantification, with which there was a 2-fold higher abundance observed following HS. This fold-change was also reflected for EEFDDQLK (at ~2.8 fold), although the absolute cpc value could not be used due to incomplete digestion of the endogenous peptide. Despite this, an unpaired t-test between the peptide cpc values for TTPSFVGFTDTER across biological replicates did not indicate significant HS-induced upregulation (p = 0.07).

### 5. Calculation of error bars for fold change between NG and HS

To determine the error for a fold change between NG and HS, we took all HS biological replicate cpc values for a single protein, and divided by the median NG cpc for the same protein to establish a set of fold changes. For the same protein, we also took the median HS cpc, and divided by each of the NG biological replicate protein cpcs, establishing a second set of fold changes. We then applied robust standard deviation and rCV calculations across both sets of fold changes and used the robust standard deviation to determine the error for the protein fold change (HS cpc / NG cpc).


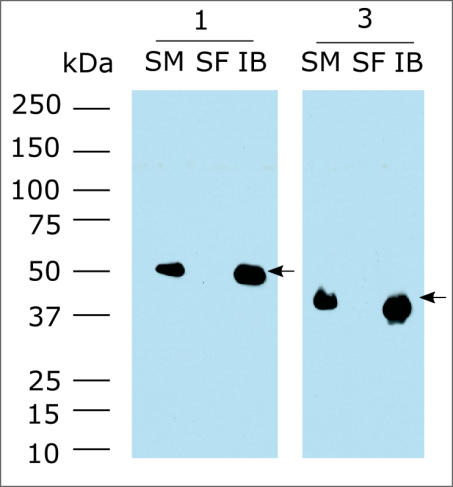

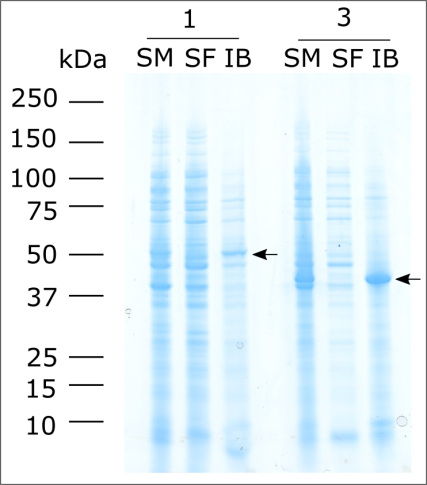


Figure S1) Extraction of ChapCAT from inclusion bodies. Expressed ChapCAT (shown are ChapCAT001 and ChapCAT003) was isolated from the inclusion bodies of *E. coli* and subject to further purification. Validation experiments were performed by western blotting with an α-His antibody. SM: starting material; SF: soluble fraction; IB: inclusion bodies. ChapCATs are indicated with an arrow.


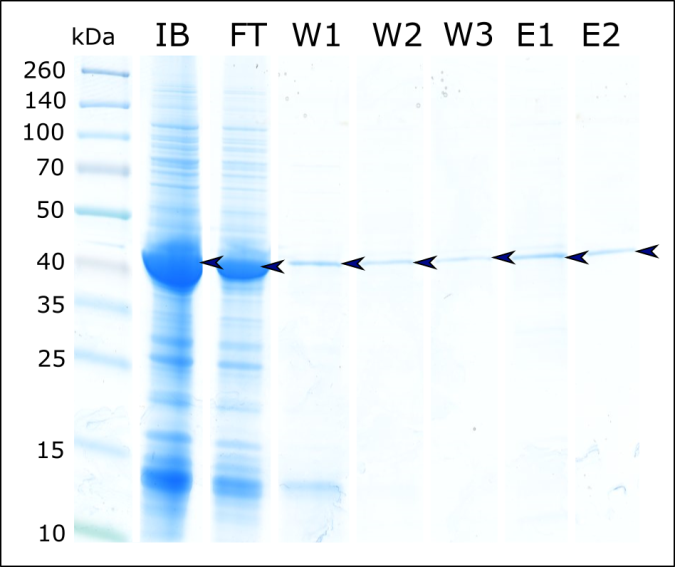


Figure S2) Purification of the ChapCAT protein from the *E. coli* inclusion bodies. Following isolation from the inclusion bodies, the ChapCAT was subject to purification via Nickel-affinity chromatography. Three wash (W) fractions were collected, containing low levels of ChapCAT. Following addition of imidazole, the ChapCAT was eluted (E) in two further fractions which were pooled and subject to dialysis and concentration.

Figure S3) Digestion Time Course. Using pseudo-first order rate kinetics, a limit peptide’s digestion progress can be estimated. With digestion completion occurring at 5 half-lives we could determine which peptides were likely to lead to incorrect quantification. We observed various digestion behaviours: A) Fast release in both standard and analyte peptide, with digestion going to completion within the time period allocated. B) Fast standard release, but slow analyte release. Both go to completion. C) Fast analyte release, slow standard release. Both go to completion. D) Slow analyte and slow standard release. Both go to completion. E) Slow analyte and standard release, without going to completion. However, progress is similar and so likely to produce accurate quantifications. F) Slow standard release, even slower analyte release without going to completion. As the accuracy for quantification is determined by the slowest release peptide, and progress of digestion is very different between the two, this peptide cannot be used for quantification, and is classified an ‘A2’.

A)

B)

C)

D)

E)

F)


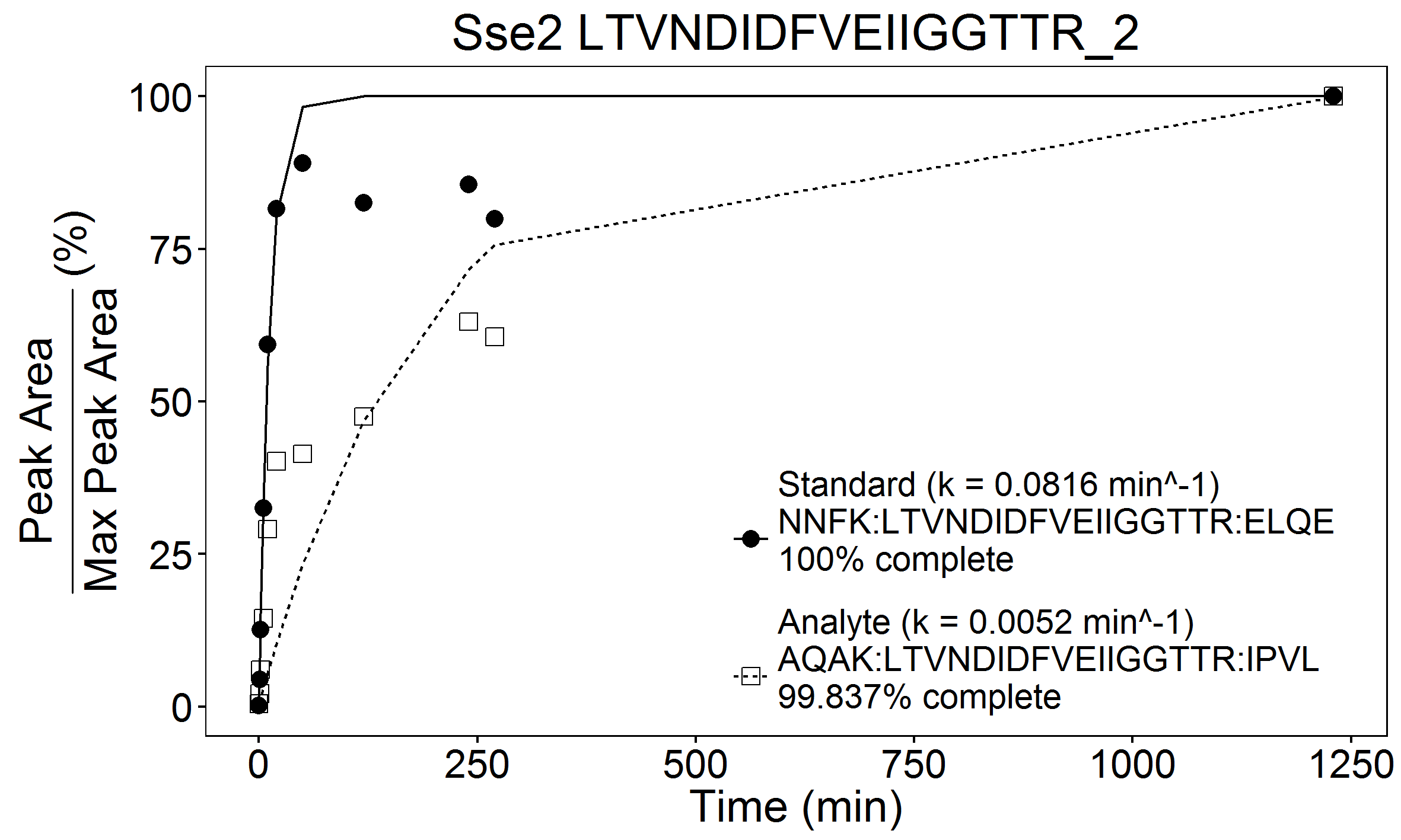

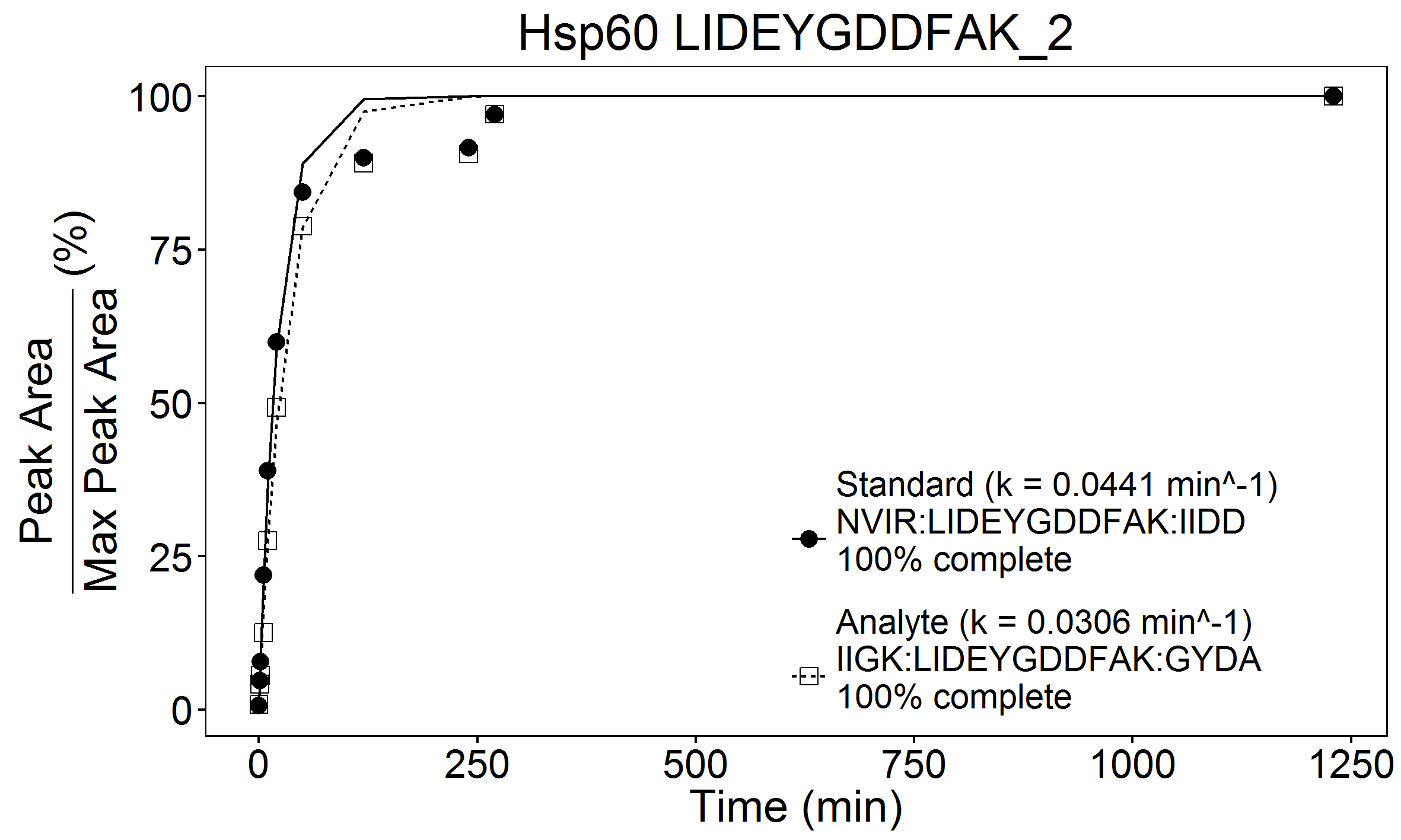


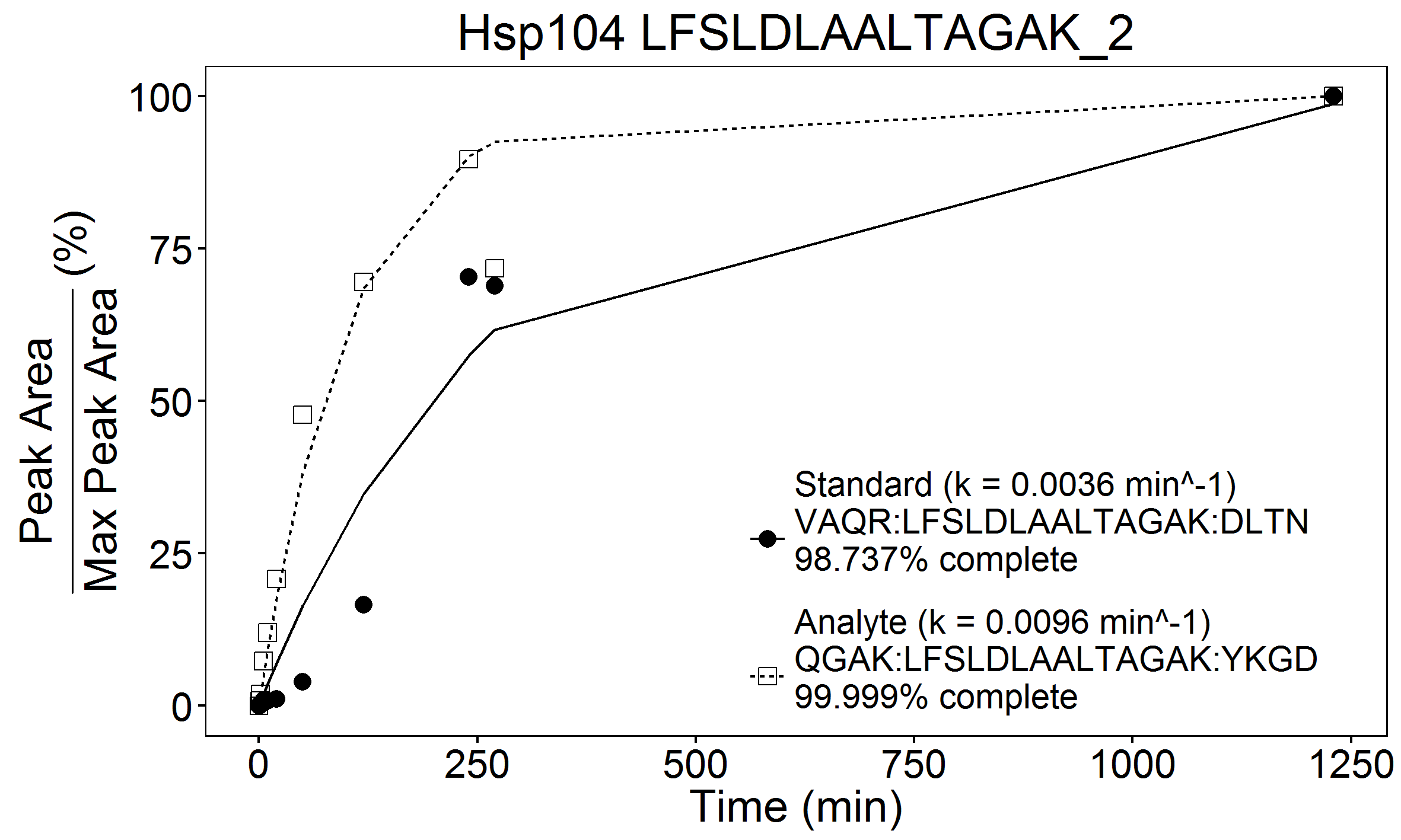

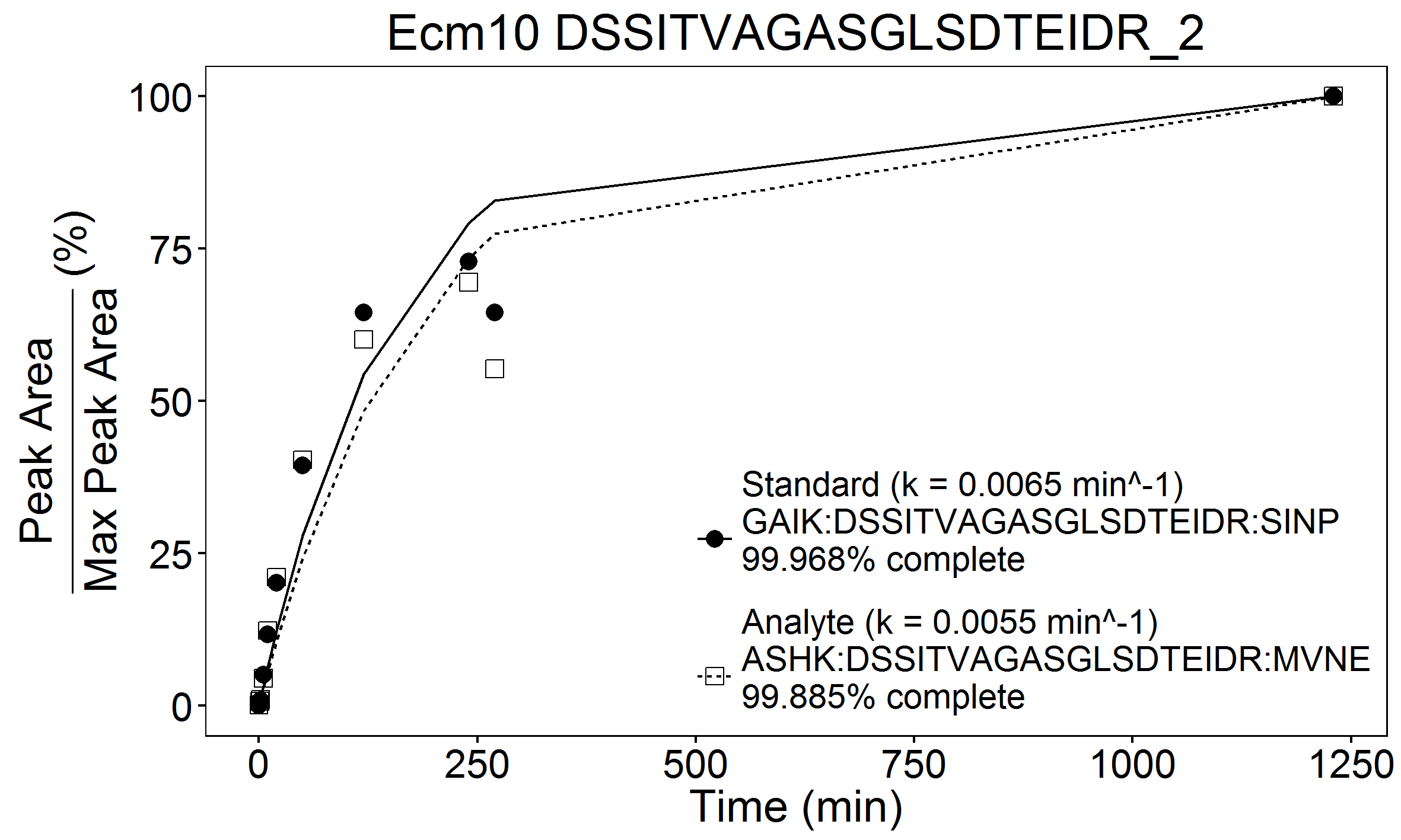


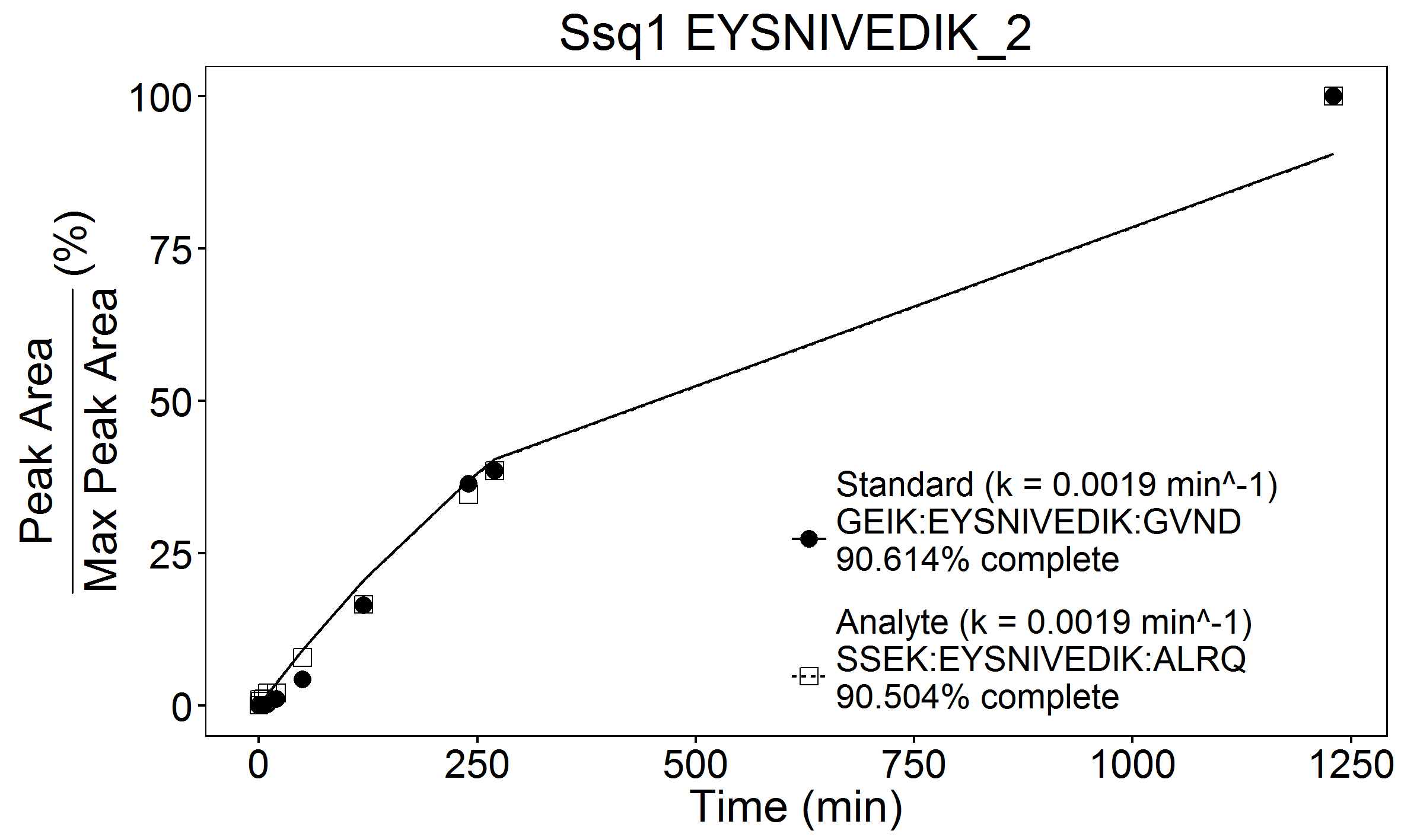


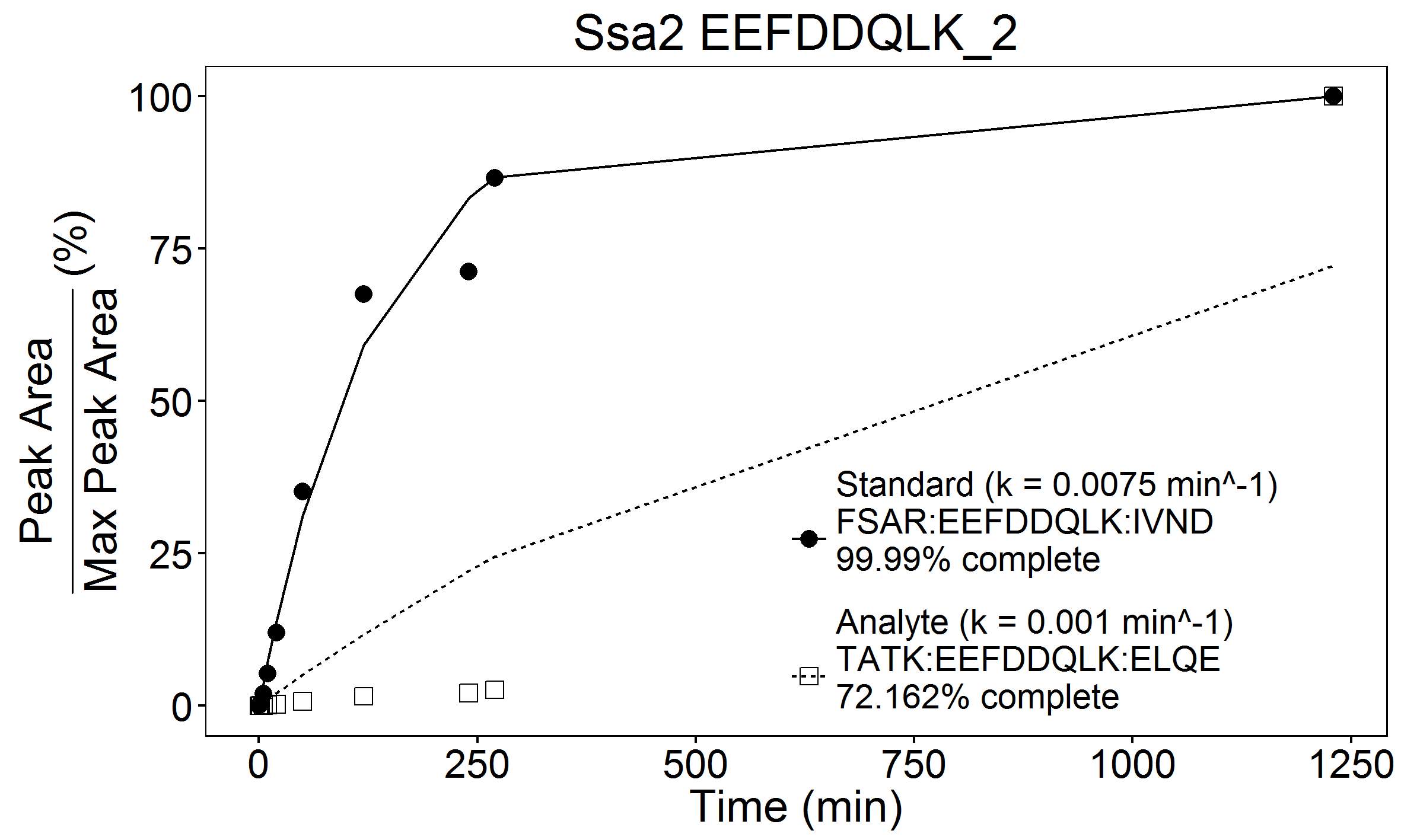


**No**

Does the peptide have an rCV of over 30?

Is it a B peptide in another condition?

**Yes**

Can it be manually curated to improve rCV?

**No**

**Yes**

**Do not use for protein quantification (A2)**

**No**

Is it the only A peptide available to use?

**Start**

**Use for protein quantification (A1)**

Does the A to B follow the same change behaviour as other peptides from the same protein?

**No**

**Yes**

**Yes**

**Yes**

Do other peptides from the same protein also have an rCV over 30?

**No**

**No**

Is there anything ‘wrong’ with the peptide?

(PTM, mProphet peak picking, transition profile, digestion, etc.)

**Yes**

**Yes**

**No**

Figure S4) Decision tree for classification of A peptides for suitability for quantification. For each peptide that was observed as both a light (yeast) and a heavy (ChapCAT) variant, quantification may be performed. However, various situations decree that erroneous quantification will follow if the Q-peptide exhibits particular traits. Such peptides were sorted into ‘A1’ and ‘A2’ classifications, and protein quantification performed as a median of all biological replicates of all ‘A1’ Q-peptides targeting a particular protein.

A)

C)

B)

E)

D)


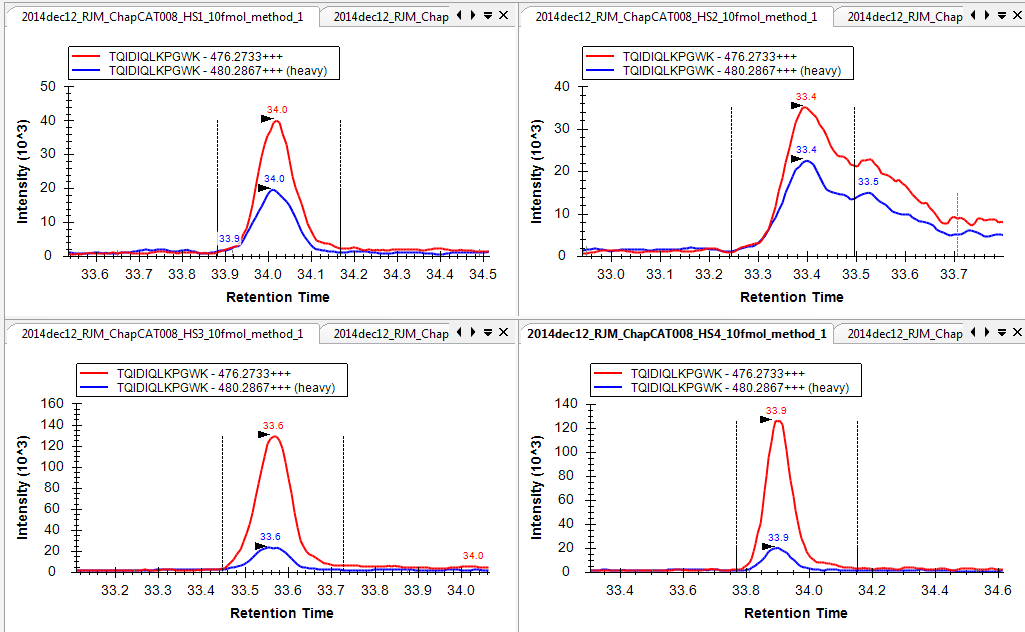

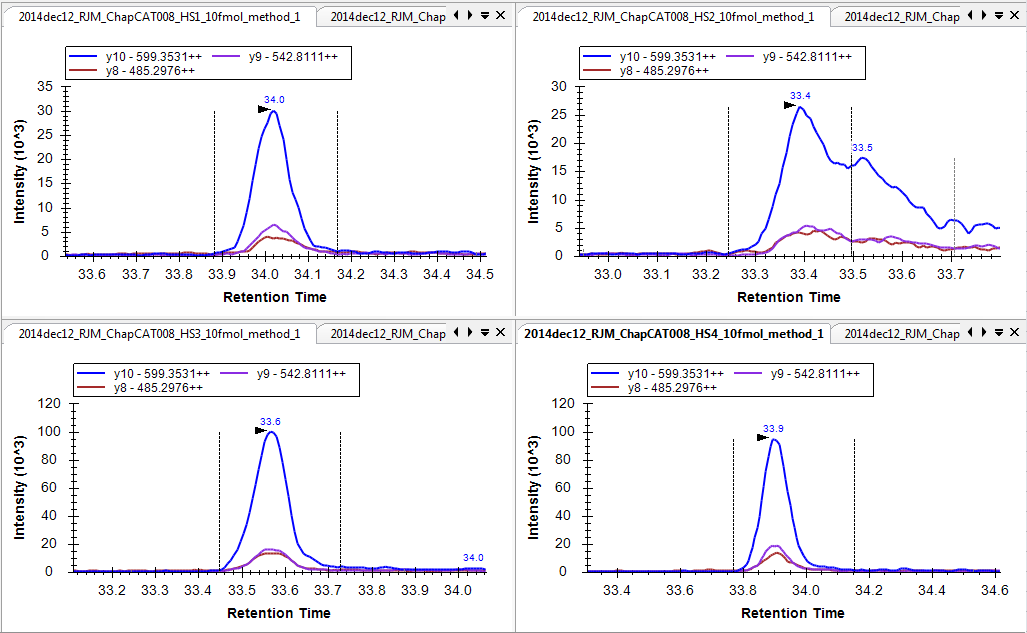

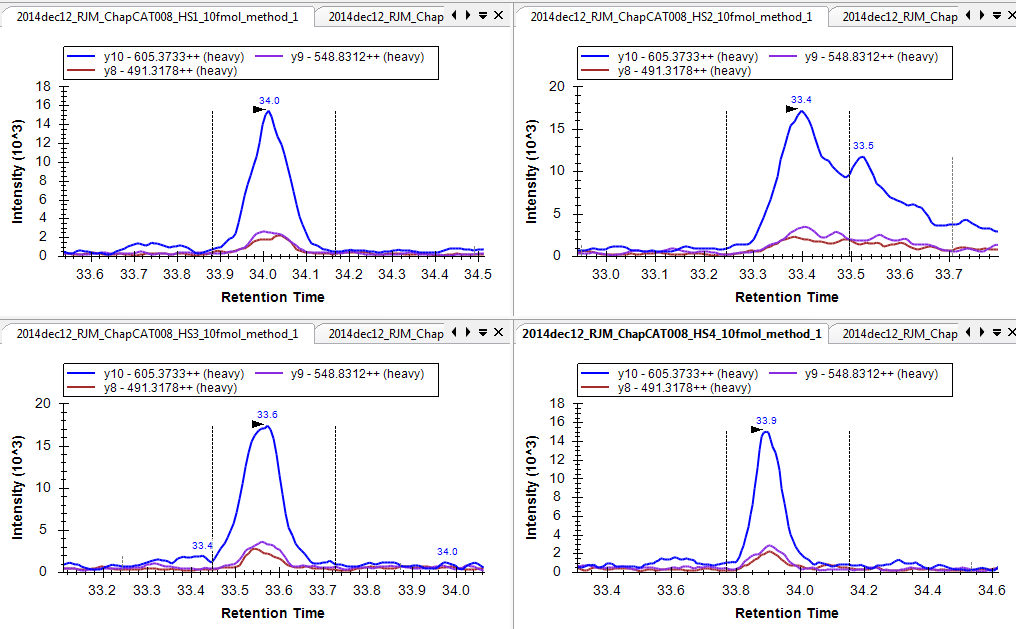

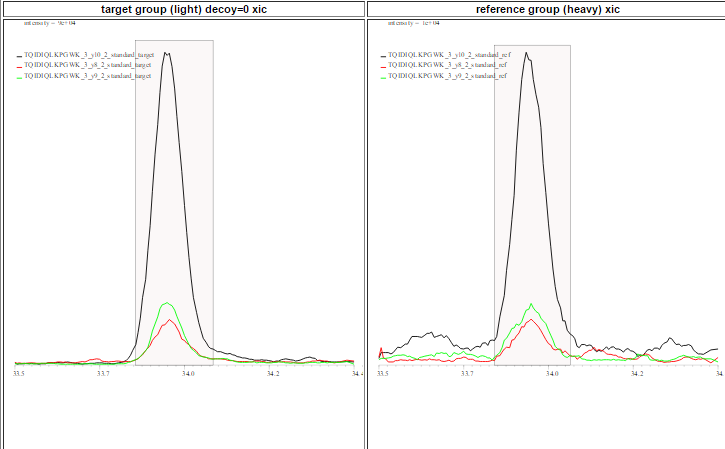


Figure S5) Classification of an ‘A’ peptide to ‘A2’. Any peptide that had a high rCV (> 30) was flagged and investigated in terms of its transition profiles, mProphet peak group selection, physicochemical properties and relationship in terms of abundance to other Q-peptides targeting the same chaperone. A) We observed the peptide TQIDIQLKPGWK to be four-fold higher in abundance than all other Q-peptides targeting the same protein, with an exceptionally high rCV of 54. B) For the heavy (ChapCAT) and light (yeast) transition profiles in Skyline we observed ChapCAT (blue) to be lower than the yeast (red) signal even at its highest concentration. C) For light (yeast) transitions we observed varying intensities and a chromatography issue in HS2. D) For heavy (ChapCAT) transitions, we observed the same chromatographic issue in HS2. E) We observed no incorrect peak group selection by mProphet (HS4 is shown).





Figure S6) Observation of the spread of data points, reflecting rCV, for each ‘A1’ Q-peptide for every chaperone. To investigate the cpc values on a per peptide basis and the effect on the target protein cpc value we observed the spread of biological replicate data points unique to each condition. We found that the rCV is not condition-dependent.


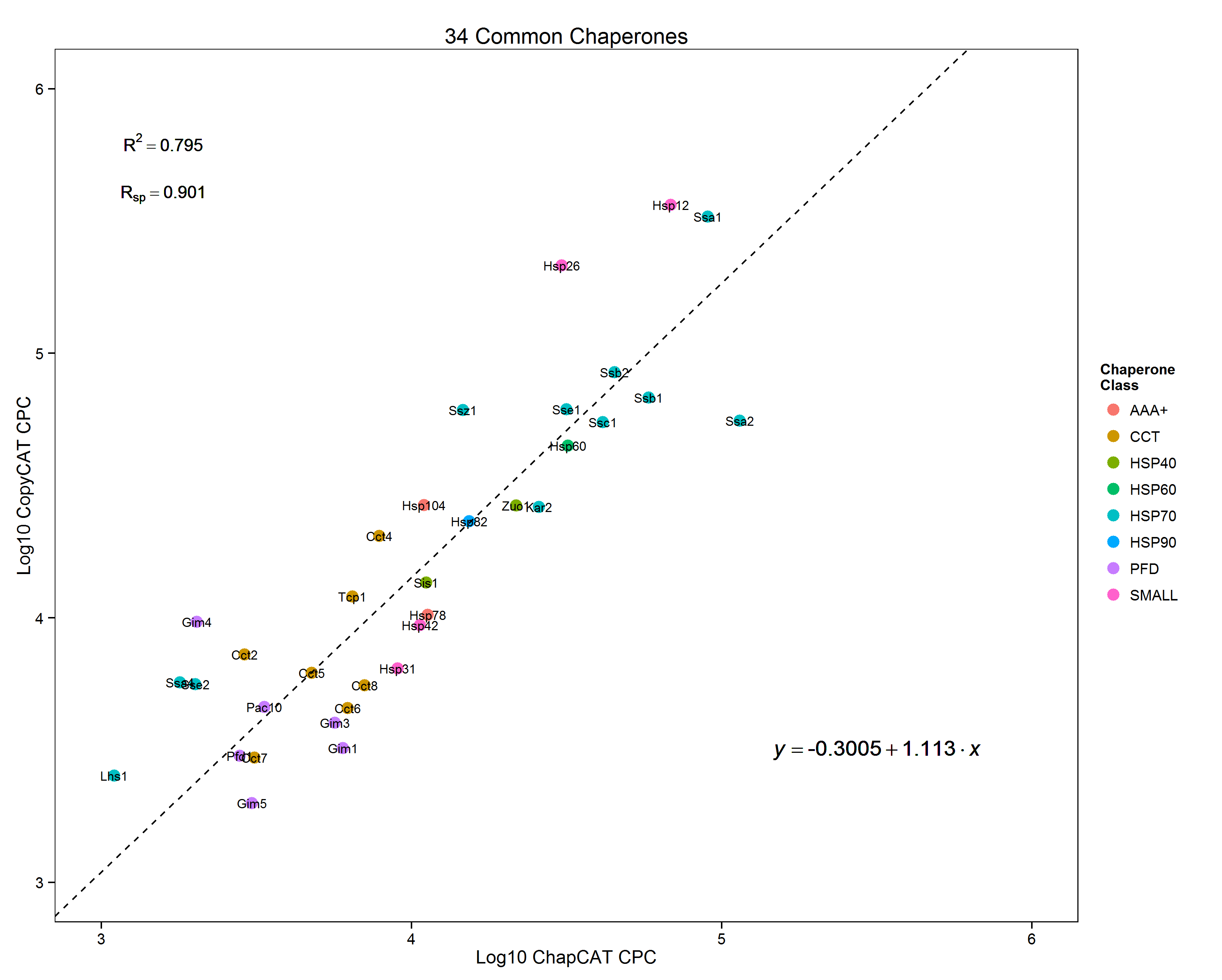


Figure S7) Comparison of protein CPC values for 34 common yeast chaperones from this study (ChapCAT) and our previously published study by Brownridge et al., (CopyCAT). Each point corresponds to the median value of 4 biological replicates for a given protein, labelled with the SGD name of its parent protein.


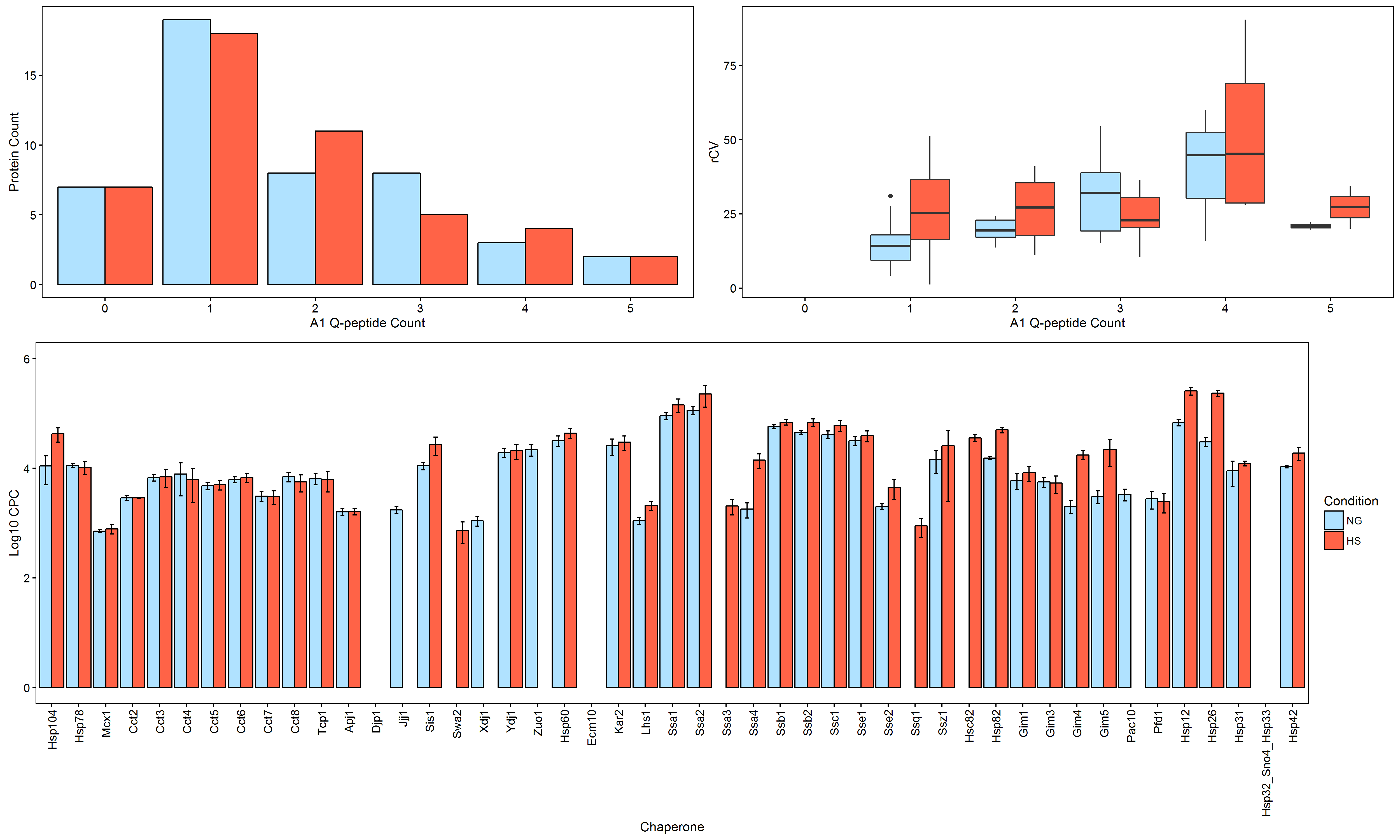


Figure S8) Absolute quantification of chaperones in NG and HS conditions.­ A) The majority of chaperones quantifications were performed using 1 or 2 ‘A1’ class Q-peptides. B) We observed a small increase in the median rCV for chaperone quantification as the number of ‘A1’ Q-peptides used for quantification increased. C) We observed upregulation for the majority of chaperones in response to HS.

A)

B)

C)

## Table S1) Expression Protocol for ChapCATs in minimal medium

Following expression testing in LB medium, the optimum expression conditions were determined. For ChapCATs that encountered difficult expression in LB medium, the addition of 10 mM benzyl alcohol was used to attempt higher levels of quantification. This was not successful for the ChapCAT010 construct and so was discontinued after initial testing.

## Table S2) Transition list for top 3 SRM ChapCAT Experiment

For each Q-peptide, where possible the top 3 transitions were determined in an unscheduled experiment. The same top 3 transitions were thus used to quantify the analyte in HS experiments.

## Table S3) Quantification on a per peptide basis

We defined limits of detection for ‘B’ peptides according to the minimum ChapCAT concentration at which the heavy ChapCAT signal is closest to a 10:1 signal-to-noise ratio. We then use this concentration to define an upper limit for the cpc value for that particular peptide for each biological replicate. For all ‘A’ peptides, the cpc value was recorded, however only biological replicates of ‘A1’ peptide values were taken into consideration when determining the final protein cpc value.

## Table S4) Digestion Time Course rate constants and half-lives

Following analysis of digestion efficiency via SRM, pseudo-first order rate constants were calculated and the digestion progress determined for every heavy (ChapCAT) and light (yeast) peptide variants observable via SRM. A reaction was deemed complete within 5 half-lives, with accurate quantification only achieved if both standard and analyte are completely digested and/or were at similar stages in their respective digestion progress.

## Table S5) Absolute quantification of chaperones in NG and HS conditions

Absolute quantification of 36 chaperones was achieved in both normal growth and heat shock conditions with a minimum of 1 Q-peptide across 3 biological replicates (where a biological replicate had not passed 1% FDR during processing by mProphet). Maximum cpc values were 114,000 and 260,000 for NG and HS respectively whilst the minimum cpc value was 700 under both conditions.

## Table S6) Label free single shot protein identifications by MaxQuant

The proteingroups.txt file from MaxQuant was filtered as described according to a minimum number of unique peptides and biological replicates in both conditions. 1644 proteins were identified and relatively quantified in both NG and HS conditions via unique and razor peptides following processing in MaxQuant with a maximal Q-value of 0.009. To compare to absolute copy per cell values for chaperones observed in SRM, we calculated the median MaxLFQ intensity for each chaperone.

## Table S7) Comparing top 10 significant protein fold changes between cpc and mod-cpc

Whilst the fold change differed between MaxLFQ SRM-normalised (‘mod-cpc’) fold changes and cpc fold changes determined via absolute quantification, in the top 10 (in order of significance) we observed 8 proteins in common that were upregulated.

## Table S8) Comparing top 10 significant protein fold changes between cpc and MaxLFQ

When we compared the top 10 relative intensity fold changes to the significantly upregulated cpc chaperone dataset, we found that 7 proteins were in common. We noted that only three chaperones are significantly upregulated in response to HS according to the MaxLFQ dataset.

## Table S9) MaxLFQ SRM-Normalised cpc values for 1644 proteins in NG and HS

Using our MaxLFQ SRM-normalised approach, we were able to provide cpc values for 1644 proteins observed in an unfractionated label free experiment in both NG and HS conditions. Each protein passed 1 % FDR against a reverse decoy identification database in both conditions. The cpc value was modelled on the median MaxLFQ value across at least three biological replicates with a non-zero value.
